# Impact of Charge Transport Layers on the Structural and Optoelectronic Properties of Coevaporated Cu_2_AgBiI_6_

**DOI:** 10.1021/acsami.5c05243

**Published:** 2025-07-08

**Authors:** Jae Eun Lee, Marcello Righetto, Benjamin W. J. Putland, Siyu Yan, Joshua R. S. Lilly, Snigdha Lal, Heon Jin, Nakita K. Noel, Michael B. Johnston, Henry J. Snaith, Laura M. Herz

**Affiliations:** Department of Physics, Clarendon Laboratory, 6396 University of Oxford, Parks Road, Oxford OX1 3PU, U.K.

**Keywords:** Cu_2_AgBiI_6_ (CABI), CuI–AgI–BiI_3_ phase space, coevaporation, impurities, lead-free photovoltaics, charge transport layers, interface engineering

## Abstract

The copper–silver–bismuth–iodide compound Cu_2_AgBiI_6_ has emerged as a promising lead-free and environmentally friendly alternative to wide-bandgap lead-halide perovskites for applications in multijunction solar cells. Despite its promising optoelectronic properties, the efficiency of Cu_2_AgBiI_6_ is still severely limited by poor charge collection. Here, we investigate the impact of commonly used charge transport layers (CTLs), including poly­[bis­(4-phenyl)­(2,4,6-trimethylphenyl)­amine] (PTAA), CuI, [6,6]-phenyl-C61-butyric acid methyl ester (PCBM), and SnO_2_, on the structural and optoelectronic properties of coevaporated Cu_2_AgBiI_6_ thin films. We reveal that while organic transport layers, such as PTAA and PCBM, form a relatively benign interface, inorganic transport layers, such as CuI and SnO_2_, induce the formation of unintended impurity phases within the CuI–AgI–BiI_3_ solid solution space, significantly influencing structural and optoelectronic properties. We demonstrate that identification of these impurity phases requires careful cross-validation combining absorption, X-ray diffraction and THz photoconductivity spectroscopy because their structural and optoelectronic properties are very similar to those of Cu_2_AgBiI_6_. Our findings highlight the critical role of CTLs in determining the structural and optoelectronic properties of coevaporated copper–silver–bismuth–iodide thin films and underscore the need for advanced interface engineering to optimize device efficiency and reproducibility.

## Graphical abstract

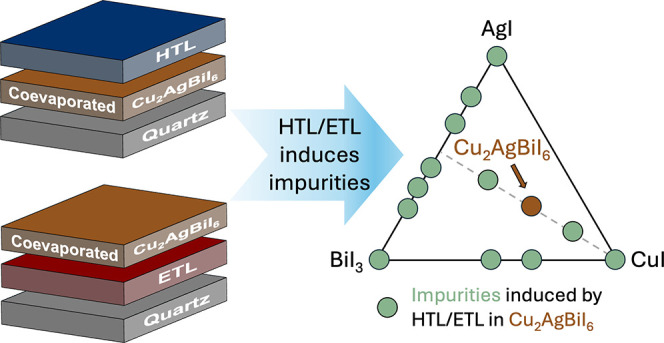

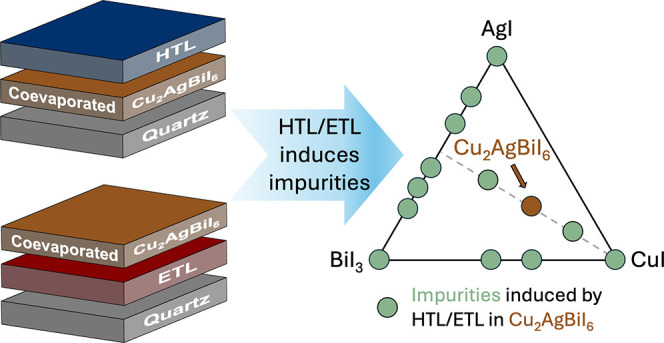

## 1 Introduction

Multijunction solar cells based on lead halide perovskites have recently achieved remarkable power conversion efficiencies (PCEs) exceeding 34% for silicon-perovskite architectures.^1^ However, their commercialization remains contingent upon the fabrication of high-quality and stable wide-bandgap perovskites (from ≈1.7 eV for perovskite-silicon to ≈2 eV for triple-junction cells).^2,3^ The well-known operational instability^4,5^ of wide-bandgap perovskite solar absorbers, together with long-standing concerns regarding the toxicity of Pb^2+^ cations,^6^ has fuelled considerable interest in all-inorganic lead-free alternatives. The substitution of Pb^2+^ with the isoelectronic pair Ag^+^/Bi^3+^ has emerged as a highly promising strategy, capable of achieving the desired wider bandgaps without introducing unstable multiple-halide alloys,^7–11^ and leading to the development of double perovskites, such as Cs_2_AgBiBr_6_.^12–15^ Silver–bismuth double perovskites exhibit excellent stability,^13,14^ moderate charge-carrier mobility around 1 cm^2^ V^–1^ s^–1^,^16^ and long charge-carrier lifetimes,^17^ resulting in promising PCEs of up to 6.37%^13^ to date. However, the indirect bandgap nature of Cs_2_AgBiBr_6_ around 2 eV, arising from the alternate Ag–Bi occupation of octahedral sites,^13,15,18^ and the recently discovered ultrafast charge-carrier localization process^16^ pose significant limitations on its maximum achievable PCE.

Such limitations have led to the exploration of a broader compositional range, focusing on the CuI–AgI–BiI_3_ phase space. Importantly, introducing Cu^+^ ions has the 2-fold aim of gaining an extra degree of chemical tunability, and increasing electronic density at the top of the valence band, thus aiming for a direct bandgap character.^9,11^ Among these, the most extensively studied Cu–Bi–I ternary compound CuBiI_4_ ^19–21^ exists as a metastable phase, decomposing back into CuI and BiI_3_ precursors at room temperature.^9^ However, ternary Ag–Bi–I compounds such as AgBi_2_I_7_, AgBiI_4_, Ag_2_BiI_5_, and Ag_3_BiI_6_ are structurally more stable and widely studied.^8,22–25^ In particular, Ag_3_BiI_6_ achieved a moderate record PCE of 5.56% through bandgap engineering via sulfide additives,^22^ but a low reported charge-carrier mobility (≈0.03 cm^2^ V^–1^ s^–1^) poses significant limitations.^7^ The quaternary compound Cu_2_AgBiI_6_ (CABI) has emerged from this compositional space as one of the most promising candidates for multijunction solar cell applications owing to its desirable optoelectronic properties with improved stability.^9,26^ CABI features a direct bandgap of ≈2 eV with a high absorption coefficient exceeding 10^5^ cm^–1^,^9,11,27^ an exciton binding energy comparable to thermal energy at room temperature,^11^ moderate charge-carrier mobilities (≈1 cm^2^ V^–1^ s^–1^) comparable to those of Cs_2_AgBiBr_6_,^27^ and extended lifetimes up to hundreds of nanoseconds.^18,28^ These properties position CABI as a strong candidate for lead-free, all inorganic top cell absorbers for tandem solar cells. Pai et al. have fabricated CABI solar cells with a modest record PCE of 2.39% by improving the film morphology using the hot-casting assisted spin-coating method on a mesoporous TiO_2_ scaffold.^18^ However, such photovoltaic device efficiency values seem surprisingly low given CABI’s favorable optoelectronic properties, raising questions on the underlying factors.

In this context, CABI-based solar cells have been recently found to suffer from limited charge transport and low current density.^9,18,28,29^ Buizza et al. have reported the presence of intrinsic charge-carrier localization processes in CABI occurring on a picosecond time scale.^27^ However, they noted that despite this process, which intrinsically lowers charge-carrier mobility, CABI still subsequently retains a decent mobility around 1 cm^2^ V^–1^ s^–1^ owing to the low energetic barriers involved in the localization process.^27^ Moreover, such ultrafast localization phenomena have also been observed in Cs_2_AgBiBr_6_ and Ag–Bi–I compounds as well,^7,16^ suggesting that the low efficiency of the state-of-the-art CABI solar cells may be attributed to extrinsic rather than intrinsic factors, potentially arising only once the material is incorporated into a device structure.

The low solubility of binary iodide precursors in the commonly used solvents (e.g., DMSO/DMF) is detrimental to the quality of solution-processed CABI thin films,^18,26^ and hence requires further optimization steps (e.g., thermal, antisolvent, additive treatments).^18,29^ Physical vapor deposition techniques have therefore been recently proposed as a promising synthetic route that enables control over thickness, uniform coating and ease of scale-up. Putland et al. deposited CABI thin films using a vapor phase coevaporation method for the first time, yet the reported PCE of the champion device only reached a modest value of 0.43%.^30^ Interestingly, the associated EQE measurements revealed negligible and imbalanced charge extraction. Given the comparable effective electron and hole masses of CABI (1.0 and 0.6 *m*_0_ respectively),^9^ such imbalanced and inefficient charge extraction has raised concerns about the quality of the interfaces between CABI and charge transport layers. The quality of the interfaces plays a critical role in the improvement of charge extraction from solar cells. For instance, wide-bandgap perovskites, typically employed as the top (sun facing) absorber layer in tandem solar cells, have yielded large *J*_sc_ values in single-junction cells when ionic additives at the charge transport layer/absorber interface had been incorporated,^31,32^ or when novel self-assembled molecular monolayers had been utilized which may enable conformal coverage and better energy-level alignment at the interface.^33^ It is therefore clear that for CABI, a similar in-depth understanding and engineering of interfaces with charge transport layers is urgently needed to unleash the potential of this promising solar absorber.^32,34,35^

In this work, we systematically investigate the impact of popular hole transport layers (HTLs), including poly­[bis­(4-phenyl)­(2,4,6-trimethylphenyl)­amine] (PTAA) and CuI, and electron transport layers (ETLs), including [6,6]-phenyl-C61-butyric acid methyl ester (PCBM) and SnO_2_, on the composition, crystallinity and morphology of coevaporated CABI and examine how these changes influence its optoelectronic properties. By using a combination of X-ray diffraction (XRD) and optical-pump terahertz-probe spectroscopy (OPTPS), we obtain comprehensive insight on CABI/charge transport layer interfaces, which are generally concealed in the conventional UV–vis absorption characterization. Notably, we reveal that while the deposition of the organic charge transport layers (i.e., PTAA, PCBM) has a minor impact on the CABI layer, inorganic charge transport layers (i.e., CuI and SnO_2_) drive the formation of impurity phases, significantly affecting the structural and optoelectronic properties of the CABI layer. Our findings shed light on the causes of underperforming coevaporated CABI solar cells and identify CABI/charge transport layer interfaces as a crucial area of development for this promising solar absorber.

## 2 Results and Discussion

We start by exploring how the electronic states in CABI are affected by the deposition of charge transport layers. In this work, we focused on CABI and solar cell “half stacks” for the best performing n-i-p solar cell configurations^18^ (i.e., CABI is evaporated on top of ETLs and HTLs are deposited on CABI without any electrodes. The materials and methods section provides full details of CABI evaporation and ETL/CTL deposition parameters). We first measured the UV–vis absorption spectra of quartz­(Q)/CABI, Q/CABI/HTL (see Figure 1a), and Q/ETL/CABI (see Figure 1b) thin films. By comparing the normalized absorption coefficient spectra of Q/CABI with their half-stack counterparts, it is evident that the absorption onsets of Q/CABI/CuI and Q/SnO_2_/CABI exhibit a red shift, while those of Q/CABI/PTAA and Q/PCBM/CABI remain relatively consistent with that of Q/CABI. A wide range of bandgaps has been reported for CABI, spanning from 1.89 to 2.13 eV.^9,11,18,27–30^ In principle, such a significant difference may stem either from variations in the composition and structure of CABI, or from different approaches for extracting bandgap values. To rule out the discrepancies arising from different data analysis techniques, we have extracted the bandgaps for Q/CABI using three widely adopted methods: the Elliott fit, inflection and Tauc methods, as shown in Figure 1c–e. The Elliott fit method generally yields higher bandgap values (see Figure 1f), followed by those determined through the inflection and Tauc methods. We attribute this to Tauc and inflection methods underestimating the bandgap values because they neglect the presence of excitonic effects and are highly sensitive to sub-bandgap defects and scattering contributions.^36^ Such contributions are crucial in CABI, whose absorption gradually onsets from around 1.8 to 2.2 eV, which is significantly broader than that of typical lead-halide perovskites.^37^ Therefore, we have chosen bandgaps extracted from Elliott fits as a more reliable metric to compare the different CABI half-stack thin films.^38^ We note that the >200 meV spread in the bandgaps reported in the literature is significantly larger than the error arising from the use of differing bandgap extraction methods (i.e., 134 meV, see Figure 1f), thus suggesting that compositional or structural changes in the bulk CABI thin film could also play a significant role. Nonetheless, the optical bandgap of 2.13 eV obtained from the Elliott fit for Q/CABI here agrees well with the bandgap of coevaporated CABI thin films reported previously, also extracted from Elliott fits to absorption onsets.^30^ Overall, our observations thus highlight the importance of considering both deposition methods and fitting techniques when determining the bandgaps.

**Figure 1:**
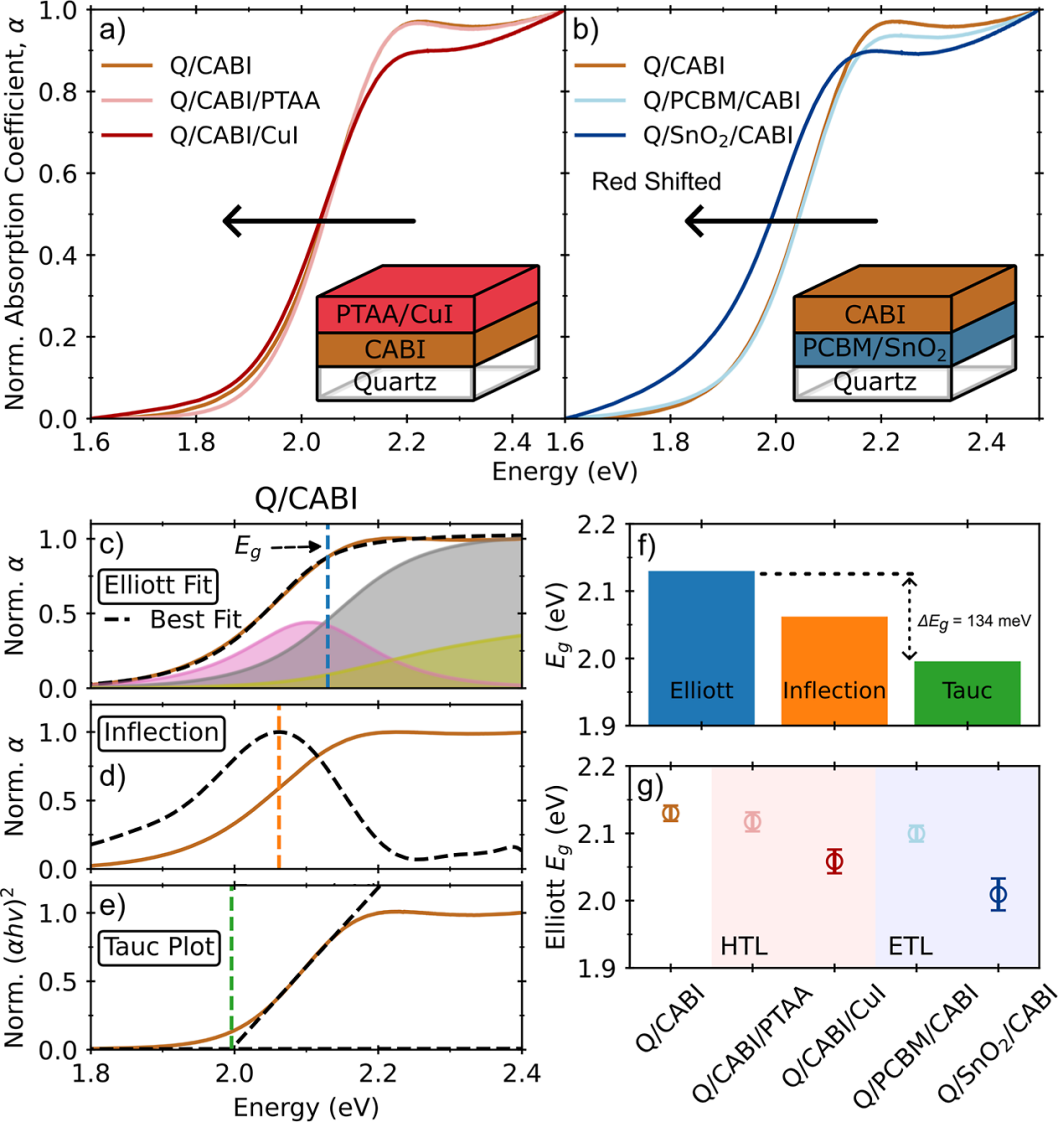
(a,b) Absorption spectra of CABI and CABI interfaced with charge transport layers. The brown solid line represents quartz­(Q)/CABI. The light red and dark red lines represent Q/CABI/PTAA and Q/CABI/CuI respectively. The light and dark blue lines represent Q/PCBM/CABI and Q/SnO_2_/CABI respectively. The insets show the half-stack architectures. (c–e) Bandgaps obtained from Elliott fit, inflection method and Tauc plot, respectively. The black dashed lines show the best fit of each method, and the vertical dashed lines represent the bandgaps obtained from each method. (f) Bandgap values obtained from each method for Q/CABI. (g) The bandgaps obtained from the Elliott fits for all thin films.

Figure 1g provides a quantitative comparison of the CABI bandgaps across the half-stack architectures, revealing a minor redshift in the bandgaps of Q/CABI/PTAA and Q/PCBM/CABI with respect to those of plain Q/CABI, and more pronounced redshifts for Q/CABI/CuI and Q/SnO_2_/CABI. Such shifts suggest the presence of non-CABI phases or structural changes in these thin films, which is further confirmed by corresponding additional absorption peaks at around 3 eV (see Figure S7), which has been recently shown to be related to the presence of CuI and AgI-rich phases.^30,39^ Furthermore, Table S2 of the Supporting Information shows that Q/SnO_2_/CABI exhibits a higher broadening parameter (γ_Abs_) associated with the absorption edge, corroborating the presence of additional energetic disorder. Such disorder is likely to originate from sub-bandgap defect states or lattice disorder induced by the impurities. We note that a nonuniform distribution of bandgaps related to impurity phases has the potential to impair charge-carrier transport and thus disrupt charge collection efficiency.

To further investigate the structural and compositional changes of the deposited thin films, we carried out XRD measurements and performed an accurate phase identification and unit cell analysis by using the Pawley fitting method.^40^ The XRD pattern of Q/CABI in Figure 2 shows distinct peaks corresponding to the (003), (012), (006), and (009) crystallographic planes of the single *R*3̅*m* space group associated with CABI (highlighted in the figure by gray vertical dashed lines), as well as a small BiI_3_ peak (marked with ○). The relative XRD peak intensities indicate that the coevaporated CABI thin film favors out-of-plane growth along the [003] direction, consistent with a previous report.^30^ The lattice parameter was obtained as *a* = *b* = 4.3167(3) Å, *c* = 20.797(2) Å, which is similar to the reported lattice parameter of the coevaporated CABI thin film (*a* = *b* = 4.3206(8) Å, *c* = 20.888(8) Å).^30^ Interestingly, interfacing the CABI film with PTAA or PCBM charge transport layers enhances the contribution of the BiI_3_ peaks, and the overlap of the BiI_3_ peak with the (003) and (009) peaks of CABI leads to the latter apparently shifting to higher diffraction angles (see Figures S14 and S15 in the Supporting Information). As expected, the Q/CABI/CuI half stack shows additional CuI peaks, marked as □, one of which dominantly overlaps with the CABI (012) peak, leading to its apparent shift to a higher diffraction angle compared to that in Q/CABI. Moreover, an additional peak related to an extra trigonal phase (*R*3̅*m*) compound is present (marked as ☆). As we discuss later, the assignment of the additional trigonal phase to a specific compound within the CuI–AgI–BiI_3_ phase space remains ambiguous because several ternary and quaternary compounds within this phase space share the *R*3̅*m* space group and exhibit similar lattice parameters. The Q/SnO_2_/CABI half stack exhibits surprisingly low XRD intensities (requiring ten times longer acquisition times to achieve a comparable signal-to-noise ratio, see the Supporting Information and Materials and Methods section for details), thus suggesting that CABI is less crystalline when deposited on SnO_2_. Moreover, peaks belonging to the binary precursors BiI_3_, CuI, and AgI (marked as △), and an additional trigonal phase are clearly observed. The observed XRD pattern suggests that the deposition of CABI on SnO_2_ leads to the incomplete formation of CABI, leaving unreacted binary halide precursors in the film, and causing unintentional formation of other trigonal ternary and quaternary phases. We reproduced and confirmed such poor film formation by undertaking several rounds of CABI deposition on SnO_2_ (see Figure S13 in the Supporting Information) with similar results.

**Figure 2:**
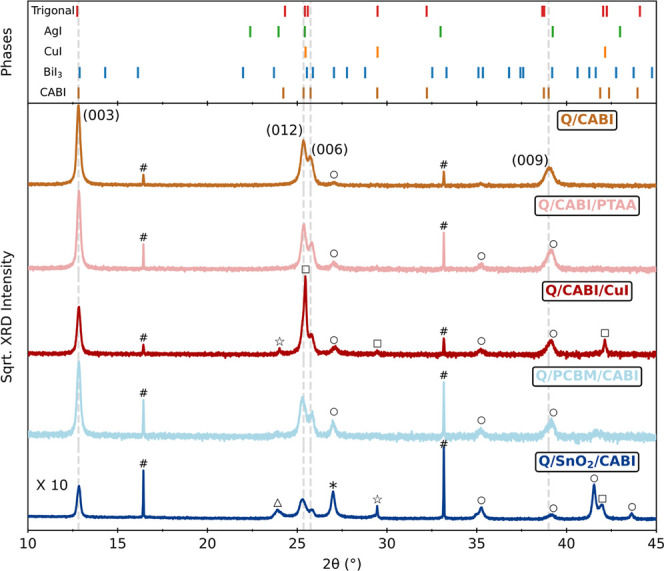
X-ray diffraction patterns (Cu Kα_1_ radiation source) of CABI films deposited on a z-cut quartz substrate (Q/CABI) and those of CABI interfaced with HTLs (PTAA and CuI) or ETLs (PCBM and SnO_2_). The XRD pattern of Q/SnO_2_/CABI was measured over a ten times longer period to obtain a comparable signal-to-noise ratio. The vertical ticks above represent the XRD peaks belonging to the additional (unassigned) trigonal phase, binary phases, and trigonal CABI phase. The gray dashed vertical lines are guides to the angular positions of (003), (012), (006), and (009) XRD peaks of the CABI phase. The symbols #, *, ○, □, △, ☆ indicate XRD peaks belonging to quartz (#), SnO_2_ (*), BiI_3_ (○), CuI (□), AgI (△), and additional ternary phases (☆) respectively.

Having identified the emergence of additional phases in nominal CABI films upon the presence of charge transport layers, we propose that the red shifts in the absorption onsets observed for the CABI half stacks (Figure 1f) derive from contributions to the absorption by these additional phases. To identify the phases responsible for this phenomenon with greater clarity, we have compiled literature information on the structure, optical bandgaps and charge-carrier mobilities at THz frequencies reported for the isolated binary, ternary and quaternary phases, as summarized in Table 1. First, we can rule out a direct influence of the AgI and CuI precursors on the lower-energy absorption onset of the half stacks because the bandgaps of AgI and CuI (2.86 and 3.1 eV respectively) are significantly higher than that of CABI. BiI_3_ has an optical bandgap slightly lower than CABI (1.77 eV vs 1.89 eV from the direct Tauc method and 2.00 eV vs 2.06 eV from the Elliott fitting). In general, the binary precursors exhibit structural properties that are clearly distinct from those of CABI, and, apart from BiI_3_, these are not dominant in the measured XRD patterns of nominal CABI films, suggesting the slightly red-shifted bandgaps for Q/CABI/PTAA and Q/PCBM/CABI are likely caused by the contribution from BiI_3_. More challenging, on the other hand, is a distinction of the CABI phase from other ternary and quaternary phases based on the XRD patterns and bandgaps, because they all share similar structures and optical bandgaps. We note that most of the ternary and quaternary phases in the CuI–AgI–BiI_3_ phase space share a trigonal unit cell with *R*3̅*m* space group and their XRD peaks could therefore overlap within the line width. Similarly, Table 1 shows that the bandgaps of the ternary and quaternary phases fall into a similar range just below the CABI absorption onset. However, determining bandgaps with a variety of methods introduces inconsistencies, obfuscating their net contribution to the observed onset. For example, the bandgap of AgBiI_4_ obtained from the direct Tauc method (1.73 eV) is lower than that of CABI (1.89 eV). However, the bandgap obtained from the Elliott fitting is larger for AgBiI_4_ (2.11 eV) than for CABI (2.06 eV). This discrepancy complicates the efforts to identify and quantify the extent to which impurity phases affect the optoelectronic properties of CABI.

**Table 1:** Summary of Structural Information, Bandgaps and THz Electron–Hole Sum Mobilities of Commonly Studied Compounds in the CuI–AgI–BiI_3_ Phase Space^c^

									THz mobility (cm^2^ V^–1^ s^–1^)	
system	compound	unit cell	space group	lattice parameters (Å)	direct Tauc bandgap (eV)	indirect Tauc bandgap (eV)	Elliott bandgap (eV)	inflection bandgap (eV)	ϕμ_deloc_	ϕμ_loc_
binary	AgI	hexagonal^41^	*P*6_3_*mc*	*a*/*b* = 4.59	2.86 ^42^	-	-	-	11 ^7^	
				*c* = 7.52						
binary	BiI_3_	trigonal^43^	*R*3̅	*a*/*b* = 7.52	N/A^44^	1.69 ^8^	2.00 ^7^	1.92 ^7^	1.4^a^ ^,^ ^7^	0.19^b^ ^,^ ^7^
				*c* = 20.72						
binary	CuI	cubic^45^	*F*4̅3*m*	*a*/*b*/*c* = 6.1	2.6,^46^ 3.0,^47^ 3.1 ^48^	N/A^48^	-	3.06 ∼ 3.11 ^49^	16 ^30^	
ternary	AgBi_2_I_7_	cubic^25^	*Fd*3̅*m* (3D)	*a*/*b*/*c* = 12.223	1.87 ^25^	1.66 ^25^	2.09 ^7^	2.06 ^7^	0.49^a^ ^,^ ^7^	0.01^b^ ^,^ ^7^
ternary	Ag_2_Bi_3_I_11_	cubic^50^	*Fd*3̅*m* (3D)	*a*/*b*/*c* = 12.1814	1.81 ^50^	1.69 ^50^	-	-	-	
ternary	AgBiI_4_	cubic^8^	*Fd*3̅*m* (3D)	*a*/*b*/*c* = 12.108	1.73 ∼ 1.80 ^8^	1.63 ∼ 1.75 ^8^	2.11 ^7^	2.03 ^7^	0.46,^a^ ^,^ ^7^ 0.7^a^ ^,^ ^7^	0.02,^b^ ^,^ ^7^ 0.07^b^ ^,^ ^7^
		trigonal^8^	*R*3̅*m* (3D)	*a*/*b* = 4.319						
				*c* = 20.600						
ternary	Ag_2_BiI_5_	trigonal^51^	*R*3̅*m* (3D)	*a*/*b* = 4.350	1.85 ^52^	1.62 ^52^	2.11 ^7^	2.02 ^7^	0.49^a^ ^,^ ^7^	0.03^b^ ^,^ ^7^
				*c* = 20.820						
ternary	Ag_3_BiI_6_	trigonal^53^	*R*3̅*m* (3D)	*a*/*b* = 4.3537	1.89 ^54^	1.83 ^54^	2.15 ^7^	1.99 ^7^	0.41^a^ ^,^ ^7^	0.02^b^ ^,^ ^7^
				*c* = 20.810						
ternary	CuBiI_4_	cubic^21^	*Fd*3̅*m* (3D)	*a*/*b*/*c* = 12.166	1.79,^21^ 1.84,^19^ 2.67 ^20^	-	-	-	-	
		trigonal^10^	*R*3̅*m* (3D)	*a*/*b* = 4.299						
				*c* = 21.060						
ternary	Cu_2_BiI_5_	trigonal^55^	*R*3̅*m* (3D)	*a*/*b* = 4.42	1.53 ∼ 1.74 ^55^	-	-	-	-	
				*c* = 20.82						
quaternary	Cu_0.4_AgBiI_4.4_	trigonal^11^	*R*3̅*m* (3D)	*a*/*b* = 4.353	-	N/A^11^	2.05 ^11^	-	0.6 ∼ 1.3^a^ ^,^ ^11^	0.06 ∼ 0.31^b^ ^,^ ^11^
				*c* = 20.791						
quaternary	CuAgBiI_5_	trigonal^10^	*R*3̅*m* (3D)	*a*/*b* = 8.634	1.94 ^18^	N/A^18^	2.06 ^11^	2.02 ^10^	0.9 ∼ 1.6^a^ ^,^ ^11^	0.13 ∼ 0.84^b^ ^,^ ^11^
				*c* = 21.140						
quaternary	Cu_2_AgBiI_6_ (CABI)	trigonal^9^	*R*3̅*m* (2D)	*a*/*b* = 4.275	1.89 ^18^	N/A^9^	2.03,^11^ 2.06,^9^ 2.13 ^30^	-	2.0,^a^ ^,^ ^30^ 3.3 ∼ 4.6^a^ ^,^ ^11^	1.5,^b^ ^,^ ^30^ 0.99 ∼ 1.73^b^ ^,^ ^11^
				*c* = 20.940						
quaternary	Cu_6_AgBiI_10_	trigonal^11^	*R*3̅*m* (3D)	*a*/*b* = 4.307	1.72 ^56^	N/A^11^	2.00 ^11^	-	7.41 ∼ 9.77^a^ ^,^ ^11^	1.09 ∼ 2.40^b^ ^,^ ^11^
				*c* = 21.171						

a THz mobility of delocalized charge carriers (ϕμ_deloc_).

b THz mobility of localized charge carriers formed on a picosecond time scale (ϕμ_loc_).

c N/A represents not applicable, and - indicates that values have not yet been reported. For the quaternary phases, THz mobilities obtained from photo-excitation with energies below 3 eV are listed to rule out contributions from CuI or AgI-rich domains.^30,39^

Interestingly, the literature values listed in Table 1 suggest that the charge-carrier mobilities (measured by THz spectroscopy) of ternary compounds and other quaternary phases differ significantly from that of CABI. Crucially, THz mobility is measured on ultrafast time scales and reflects local length-scales, thus making these measurement less sensitive to any presence of long-range grain boundaries. Therefore, we find that THz mobilities are a valuable metric for distinguishing and cross-referencing between different phases present in nominal CABI films. For example, the trigonal Ag–Bi–I ternary phases exhibit THz mobilities that are at least four times lower than those of CABI (≈0.5 vs ≈ 2 cm^2^ V^–1^ s^–1^). Although the THz mobilities of CuBiI_4_ and Cu_2_BiI_5_ have not been measured yet, the reported Hall mobility of 110 cm^2^ V^–1^ s^–1^ for CuBiI_4_ suggests that its THz mobilities may also be significantly higher than that of CABI.^20^ As recently demonstrated by Buizza et al., THz mobilities of the quaternary phases Cu_4*x*_(AgBi)_1–*x*_I_4_ increase with increasing Cu content.^11^ This trend has been attributed to the substantial contributions from the enhanced curvature of the valence band maximum of the Cu-*d* electronic states.^9^ As a result, a significant difference in the THz charge-carrier mobilities facilitates the distinction of the lower Cu-content Cu_0.4_AgBiI_4.4_ and the higher Cu-content Cu_6_AgBiI_10_ from CABI. CuAgBiI_5_ has similar bandgap and THz charge-carrier mobility to those of CABI, but it can be distinguished from the XRD patterns since it has significantly different lattice parameters associated with a large trigonal unit cell.^10^ Overall, the identification of the CABI phase can therefore be accomplished with greater confidence when supported by THz mobility measurements as an additional metric.

Having established that the THz mobilities offer useful complementary information about different phases within the CuI–AgI–BiI_3_ phase space, we extract the THz mobilities of charge carriers within the nominal CABI films in our half stacks from fluence-dependent OPTPS measurements. OPTPS measures the differential change in the transmitted THz electric field amplitude before and after photoexcitation (−Δ*T*/*T*), which is proportional to the transient photoconductivity of the material, with subpicosecond temporal resolution (see the Supporting Information for further experimental details). We note that the half stacks were excited at a wavelength of 530 nm (2.34 eV photon energy) in order to selectively excite the CABI solar absorber layer^30,48,57^ rather than the HTLs or ETLs. The photoconductivity transients of CABI on quartz (Figure 3a) exhibit a fluence-independent ultrafast decay within a couple of picoseconds, followed by a slower, long-lived fluence-dependent decay (see Figures S18–S22 in the Supporting Information for OPTP transients for all half stacks). To describe such dynamics, the early time photoconductivity transients from −2 to 10 ps were fitted with a two-level mobility model previously employed to describe the ultrafast localization processes in Cs_2_AgBiBr_6_, Cu_4*x*_(AgBi)_1–*x*_I_4_, (AgI)_*x*_(BiI_3_)_*y*_ and other silver–bismuth based materials.^7,11,16,58^ As indicated by the schematic in Figure 3b, this model assumes that charge carriers are initially photoexcited forming a delocalized, large-polaron state with mobility μ_deloc_ and population *n*_deloc_. Within about a picosecond after photoexcitation, however, charge carriers localize into a small-polaron state with reduced mobility μ_loc_ and population *n*_loc_ with localization rate given by *k*_loc_. Lastly, the localized electrons and holes recombine back to the ground state with the recombination rate *k*_R_. By solving the coupled ordinary differential equations governing the two states and globally fitting solutions to the fluence-dependent OPTP transients, the mobilities associated with each state can be independently calculated. A detailed mathematical description of the model can be found in the Supporting Information. To improve the accuracy of extracted parameters governing the early dynamics, *k*_R_ is fixed to the values obtained from the conventional model describing trap-mediated and radiative recombination, fitted to the long decay after 10 ps across the range of excitation fluences (see Figures S20, S21 and Table S4 in the Supporting Information). As shown in Figure 3a, the excellent agreement of the best fit (black dashed lines) with the experimentally determined photoconductivity suggests that the two-level mobility model describes the charge-carrier dynamics of CABI well.

**Figure 3:**
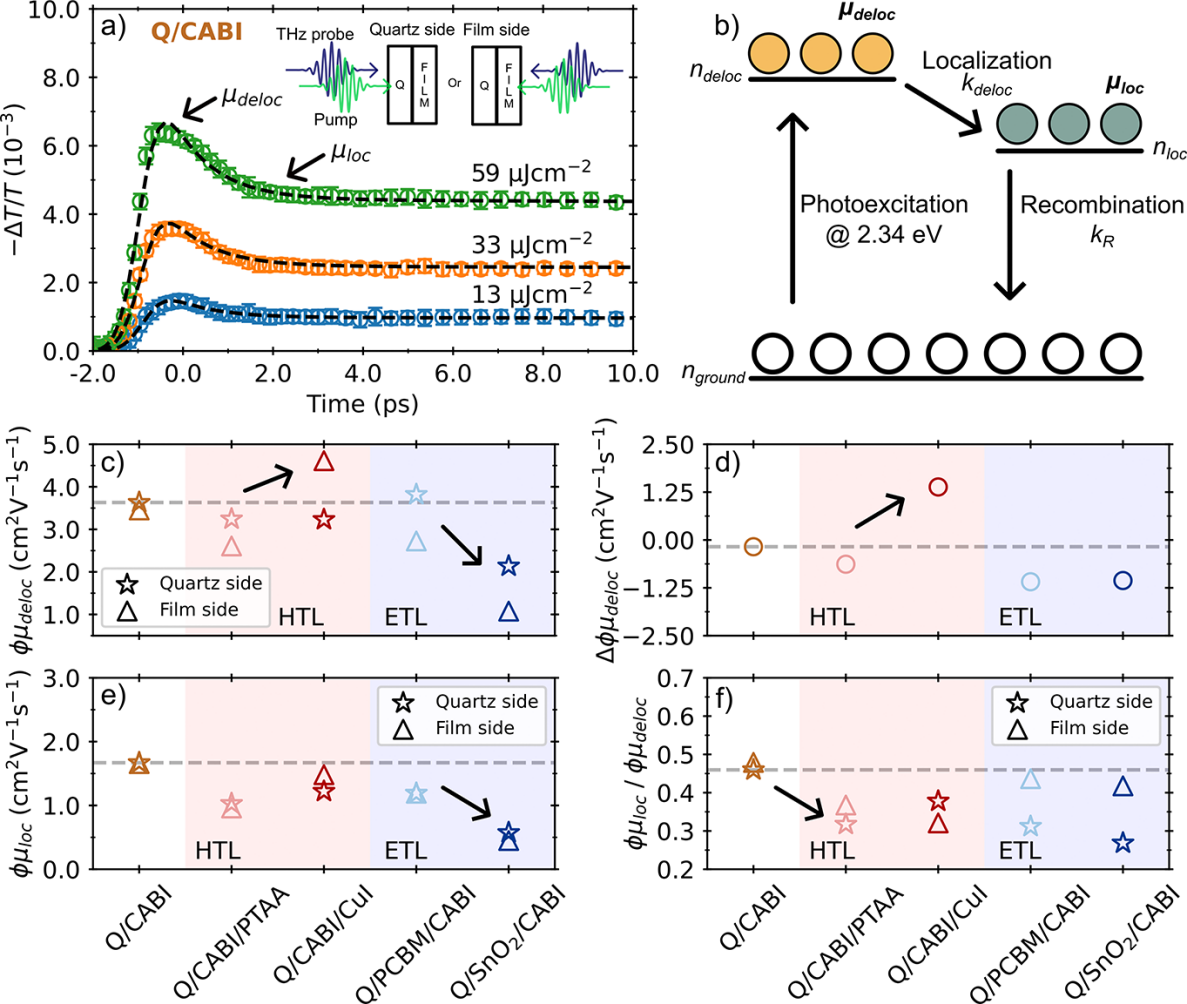
(a) OPTPS photoconductivity transient of quartz­(Q)/CABI excited from the quartz-substrate side with the three excitation fluences of 13, 33, and 59 μJ cm^–2^. The black dashed lines show the best fits of the two-level mobility model. The inset shows the two different excitation conditions, i.e. either from the side of the quartz substrate (left, “Quartz side”) or from the side containing the CABI and potentially ETL or HTL films (right, “Film side”). (b) Schematic diagram illustrating the two-level mobility model. (c) Effective delocalized electron–hole sum mobilities (ϕμ_deloc_) at THz frequencies determined following quartz-side and film-side illuminations. (d) Difference between the delocalized mobility values determined for film-side and quartz-side illumination (Δϕμ_deloc_ = ϕμ_deloc_ @ film side-ϕμ_deloc_ @ quartz side). (e) Effective localized electron–hole sum mobilities (ϕμ_loc_) at THz frequencies, determined following quartz-side and film-side illumination. (f) Ratio of localized THz mobilities to delocalized THz mobilities (*R* = μ_loc_/μ_deloc_). For (c–f), values for CABI thin films coated with HTLs are colored with a light red background and those deposited on ETLs are colored with a light blue background. The gray dashed horizontal lines are guides to the eye allowing comparison of mobilities with the value for Q/CABI. The error bars are contained within the extent of the data markers and reported in Table S4 in the Supporting Information.

We are further able to selectively probe THz mobilities at the CABI/charge transport layer through side-dependent illumination, either from the quartz substrate side or from the side of the deposited films. Because the penetration depth at the 2.34 eV excitation energy is very short (≈80 nm compared to the ≈250 nm thickness of CABI, see Figure S6 in the Supporting Information), such side-selective excitation thus allows us to determine charge-carrier mobilities in CABI either far from or close to the buried interface, and to deduce possible compositional and morphological variations along the depth profile.

The extracted THz mobility of delocalized charge carriers (ϕμ_deloc_), obtained from the initial THz photoconductivity (−Δ*T*/*T* = 0 ps), of Q/CABI is ≈3.5 cm^2^ V^–1^ s^–1^, which is in a good agreement with the literature value in Table 1. While THz mobilities probe local lengths scales and are therefore dominantly affected by intrinsic effects such as electron–phonon interactions, they may also be influenced by extrinsic factors such as extended disorder, poor morphology and crystallinity caused by the impurity phases.^59^ For Q/CABI, we observe similar ϕμ_deloc_ values following quartz-side and film-side illumination, which suggests that the composition, crystallinity and morphology of CABI near both interfaces are similar.

For the half stacks, on the other hand, Figure 3c reveals significant differences between quartz-side and film-side excitation, arising from differences in CABI film quality near the interface with the HTL or ETL. For Q/CABI/PTAA, Q/PCBM/CABI, and Q/CABI/CuI, illumination from the quartz side reveals delocalized charge-carrier mobilities that are comparable to or slightly lower than that of Q/CABI, suggesting that the impact of the minor BiI_3_, CuI, and trigonal impurity phases identified from the XRD patterns is minor on the quartz side. However, for illumination from the film side, Q/CABI/PTAA and Q/PCBM/CABI exhibit ϕμ_deloc_ values that are noticeably lower than that of Q/CABI. This trend correlates well with the enhanced BiI_3_ impurity peaks present in XRD patterns and the red-shifted absorption onsets, while changes in the morphologies are not apparent from the SEM images (see Figure S1 in the Supporting Information for top-down SEM images and further discussions). We postulate that BiI_3_ impurities located near the top interface of CABI decrease the value of ϕμ_deloc_ because they may act as scattering sites with enhanced disorder and defects. For Q/CABI/CuI on the other hand, a higher value of ϕμ_deloc_ is recorded, in particular for excitation from the “film side” which leads to generation of charge carriers near the CABI/CuI interface, indicating that the deposition of CuI significantly influences the optoelectronic properties near this interface. Although the observed increase in the delocalized mobility may be attributed to passivation effects leading to improved interfacial quality, distinct changes in the XRD pattern of Q/CABI/CuI suggest that the composition of CABI is altered upon the deposition of CuI. By considering the XRD patterns and the observed bandgap redshift, which are compatible with trigonal Cu_6_AgBiI_10_,^56^ we propose that the observed increase in ϕμ_deloc_ arises from the formation of a Cu-rich Cu_4*x*_(AgBi)_1–*x*_I_4_ phase near the CABI/CuI interface, which is known to exhibit comparatively higher mobilities (see Table 1). The effect of CuI is further highlighted in Figure 3d where we present the difference in ϕμ_deloc_ extracted from film-side and quartz-side measurements (Δϕμ_deloc_). We find that this difference only has a positive value for the Q/CABI/CuI half stack, suggesting that deposition of CuI can be an effective optimization strategy to increase charge-carrier mobility at the expense of modifying the stoichiometry of CABI near such interfaces. Finally, for Q/SnO_2_/CABI half stacks we observe Δϕμ_deloc_ values significantly lower than those for Q/CABI, most likely linked with the presence of binary and additional ternary phases in CABI evaporated on SnO_2_, as evidenced from XRD. While the wide-bandgap binary phases (AgI and CuI) cannot be excited by our excitation pulses, the observed poor mobility values can in principle originate from BiI_3_, other low-mobility trigonal phases, or poor film morphology. Overall, these results strongly suggest that the presence of an SnO_2_ layer induces an incomplete reaction of the precursors which severely lowers the charge-carrier mobility associated with the CABI layer in the Q/SnO_2_/CABI half stacks. The SEM image recorded for this half stack further confirms the poor morphology of the CABI layers evaporated on SnO_2_, showing discontinued and incomplete grains over a long range (see Figure S1 in the Supporting Information for top-down SEM images of the Q/SnO_2_/CABI half stack). Similar to the case of Q/PCBM/CABI, the excitation-side dependence of the extracted mobility value (Figure 3c) suggests that the growth of CABI films on SnO_2_ particularly favors the formation of impurity phases at the top interface. Therefore, such imperfect morphology of CABI on SnO_2_ appears not to dissipate with increasing film evolution and growth.

Finally, we observe that charge-carrier mobilities determined for small polarons formed after the initial localization step (ϕμ_loc_, obtained from the THz photoconductivity after ≈2 ps), generally follow a similar trend to that discussed above for the initial large-polaron mobilities as shown in Figure 3e. Therefore, the changes in composition, morphology and crystallinity clearly affect the delocalized state as well as the localized state that dominates during charge-carrier diffusion and extraction.

Interestingly, the difference in the values of ϕμ_loc_ determined for quartz-side and film-side illuminations is not as pronounced as it is for the ϕμ_deloc_. This effect is unlikely to derive from electron- or hole-transfer from CABI to ETL or HTL layers because the low recorded mobilities prevent significant charge-carrier diffusion to the interface within the first few picoseconds. We note that in principle, small-polaron transport is more susceptible to disorder owing to its thermally activated nature.^60^ However, the observed mobility values arise from an ensemble of phases (i.e., CABI and additional binary/ternary phases), some of which are not being photoexcited (e.g., binary AgI, CuI) and others exhibiting higher degree of localization, thus making contributions difficult to disentangle. To quantify such effect, the ratio of ϕμ_loc_ and ϕμ_deloc_ (*R* = ϕμ_loc_/ϕμ_deloc_) allows us to quantify the overall impact of localization on the mobility retention considering the effect of the impurity phases. For example, the case of *R* = 1 denotes full retention of the initial large-polaron mobility, while *R* = 0 represents full mobility loss (ϕμ_loc_ = 0) after the first few picoseconds. As presented in Figure 3f, CABI on quartz shows *R* ≈ 0.48, i.e. it retains about half of its mobility after localization. However, when CABI is interfaced with charge transport layers, significantly less mobility is retained after localization, i.e. lower *R* values are observed (Figure 3f). Such effects may derive from the presence of impurity phases which have been reported to exhibit greater degree of localization (lower *R* values), such as BiI_3_ (*R* ≈ 0.13), (AgI)_*x*_(BiI_3_)_*y*_ (*R* ≈ 0.06), and Cu-rich Cu_6_AgBiI_10_ (*R* ≈ 0.14).^7^ As a result, ϕμ_loc_ values are lower for all half stacks compared to Q/CABI. Such reduction in mobility retention is particularly detrimental for photovoltaic device performance, given that the charge-carrier mobility ϕμ_loc_ recorded after the picosecond localization process ultimately determines the charge-carrier diffusion length for a given charge-carrier lifetime, and therefore the feasibility of extraction.

Overall, based on multiple complementary experimental approaches, we reveal that coevaporation of CABI onto an inorganic electron transport layer (i.e., SnO_2_) is far more challenging, leading to degraded optoelectronic and transport properties, compared to deposition on organic electron transport layers (i.e., PCBM). Similar challenges have been observed in the more advanced field of coevaporated lead halide perovskites. Patel et al. have observed the formation of amorphous MAPbI_3_ following evaporating on compact TiO_2_ caused by a lattice mismatch at the interface, leading to poor charge collection efficiency and extensive charge recombination, while high-quality crystalline MAPbI_3_ films were formed with PCBM.^61^ Our findings suggest that in a similar way, CABI growth may be impeded on inorganic transport layers owing to the lattice mismatch between tetragonal SnO_2_ and trigonal CABI.^9,62^ This mismatch likely promotes island growth and therefore facilitates nonuniform grain growth, ultimately leading to incomplete precursor reaction.^63^ In addition, the large CuI–AgI–BiI_3_ phase space available makes quaternary CABI more prone than ternary MAPbI_3_ to the formation of unwanted secondary phases. Similarly, the deposition of hole transport layers (i.e., PTAA and CuI) onto CABI induces the formation of impurity phases such as BiI_3_ and Cu-rich Cu_4*x*_(AgBi)_1–*x*_I_4_ phases, which somewhat impedes transport in CABI, though this effect seems to be less severe. Crucially, these observations may explain the origin of the poor charge extraction and low reproducibility reported for solar cells incorporating coevaporated CABI absorber layers.^30^ Therefore, our results indicate the urgent need for the development of advanced interface engineering approaches enabling optimized CABI crystal growth on inorganic surfaces and facilitating the deposition of hole transport layers without disrupting CABI. For instance, Yan et al. have recently addressed analogous issues for coevaporated FA_0.9_Cs_0.1_PbI_3–*x*_Cl_*x*_ growth by introducing an ultrathin templating layer at the charge transport layer/perovskite interface prior to the deposition of the active layer.^64^ Furthermore, the optimization of solvents for spin-coated charge transport layers^65,66^ and deposition of thin buffer layers^67,68^ may help to prevent the interaction of charge transport layers with CABI.

## 3 Conclusions

In conclusion, we have systematically identified the compositional, structural and morphological changes that occur when CABI is interfaced with charge transport layers and have investigated the resulting impact on its optoelectronic properties. Our findings reveal that the presence of charge transport layers induces the unintentional formation of impurities within the CuI–AgI–BiI_3_ phase space, which in turn significantly affect the overall optoelectronic properties of CABI layers within such half stacks. Organic transport layers such as PTAA and PCBM induce minor BiI_3_ impurity phases, leading to small red shifts in the absorption edge and reduction in the THz electron–hole mobilities. On the other hand, deposition of CuI leads to the formation of a Cu-rich Cu_4*x*_(AgBi)_1–*x*_I_4_ phase near the CABI/CuI interface, associated with an increase in the initial THz mobility, which is however not sustained after picosecond charge-carrier localization. Additionally, controlling the CABI phase at an interface with CuI may be challenging, opening the door to decreased reproducibility. The deposition of CABI on SnO_2_, on the other hand, is associated with an incomplete reaction of the binary phase precursors and formation of other trigonal phases within the CuI–AgI–BiI_3_ phase space, causing a significant decrease in the THz mobility. This finding may thus explain the previously reported poor reproducibility and low charge collection efficiency of photovoltaic n–i–p devices based on CABI coevaporated on SnO_2_ as the electron transport layer.^30^ Borrowing wisdom from the extensive field of lead halide perovskites, we predict that such inorganic interfaces can however be improved with the development of tailored interface engineering approaches, unleashing the full potential of CABI-based solar cells. Overall, our findings identify phase control at the charge–extraction interface as a crucial area of development for CABI solar absorbers.

## 4 Materials and Methods

### 4.1 Fabrication of Quartz/Cu_2_AgBiI_6_

Prior to the vapor-phase deposition of Cu_2_AgBiI_6_ (CABI), a tooling factor for each precursor was calculated to correct the divergence between the true evaporation rate and the rate measured by the QCM. For this purpose, 100 nm (as measured on the SQC-310 controller) of each precursor was deposited on 30 × 30 mm glass substrates (Lumtec, product number LT-G000) and the thickness was measured using a Veeko Dektak 150 profilometer. A new tooling factor was calculated using eq 1.

1 $$tooling\;factor=default\;tooling\;factor\times \frac{thickness\;\left(Dektak\right)}{thickness\;\left(QCM\right)}$$

The thickness was later verified by using the cross-sectional SEM images as shown in Table S1 in the Supporting Information. Detailed CABI vapor-phase deposition parameters are provided together with the information on the fabrication of the various half-stack samples below. We note that Putland et al. have previously shown that for identical vapor-phase deposition protocols for CABI, subsequent annealing of the CABI films led to the formation of additional CuI peaks in the XRD patterns and poor morphology with smaller grains and increased pinhole density.^30^ For this reason, we have employed unannealed CABI thin films for our study, in order to minimize the amount of impurity phases included.

Thin films used for characterization were evaporated onto z-cut quartz substrates (UQG, 13 mm diameter × 2 mm thickness) that were first prepared by sequentially sonicating the substrates in 200 mL of Decon90 and DI water (2% concentration) (Milli-Q IQ), 200 mL of DI water (Milli-Q IQ), 200 mL acetone (ACS reagent, ≥99.5%, Sigma-Aldrich), and 200 mL isopropanol (anhydrous, 99.5%, Sigma-Aldrich) for 15 min each, then O_2_ plasma cleaned for 10 min (Diener electronics, Model: Pico), and UV-Ozone cleaned for 15 min (Jelight Company Inc., Model: 30-220) prior to deposition of Cu_2_AgBiI_6_.

Thin films of Cu_2_AgBiI_6_ were fabricated by evaporating under vacuum (BOC Edwards Auto 306) in codeposition bismuth­(III) iodide (Alpha Aesar Puratronic, 99.999%), silver­(I) iodide (Alpha Aesar Premion, 99.999%), and copper­(I) iodide (Alpha Aesar Puratronic, 99.998%) precursors from three separate, 2.4 cm^3^ alumina crucibles and thermal sources. The crucibles and sources were custom-made by Moorfield Nanotechnology to fit the dimensions of the evaporation chamber. The precursors were heated to the temperature corresponding to the following evaporation rates: CuI, AgI, BiI_3_ = 0.33 Å s^–1^ (370 °C), 0.18 Å s^–1^ (475 °C), and 0.50 Å s^–1^ (230 °C), respectively, and deposited until a Cu_2_AgBiI_6_ film with a thickness of (250 ± 20) nm was reached, as measured by a Veeko Dektak 150 profilometer. The rates were measured using three quartz crystal microbalances (QCM) positioned off center to each source’s vapor cone and an Inficon SQC-310 deposition controller.

All depositions were carried out under vacuum (∼2 × 10^–6^ mbar). The substrates were protected during the heating and cooling process by a mechanical shutter, and the substrates were rotated during deposition to improve surface coverage. No intentional substrate heating was applied. However, the substrates reached a maximum temperature of approximately 60 °C during codeposition due to heat transfer from the sources. The temperature of the substrates was measured using RS Electronics PRO nonreversible temperature sensitive labels (RS Stock No.: 779-9779).

### 4.2 Fabrication of Quartz/Cu_2_AgBiI_6_/PTAA

Cu_2_AgBiI_6_ was deposited on quartz as above. The hole transport layer poly­[bis­(4-phenyl)­(2,4,6-trimethylphenyl)­amine] (PTAA) (99.99%, Flexink) was prepared by mixing PTAA in toluene (anhydrous, 99.8%, Sigma-Aldrich) (10 mg/mL). Prior to deposition, the PTAA solution was filtered using a 0.22 μm PTFE filter (13 mm diameter, Gilson Scientific Ltd.). To deposit the layer, 150 μL was statically spin coated in ambient air at 4000 rpm spin speed, 2000 rpms^–1^ ramp rate, for 30 s. PTAA films were not annealed. The ambient air temperature and humidity were not measured for each deposition, but the cleanroom where it was carried out ranges in temperature between 18 and 25 °C with a relative humidity between 20 and 50%.

### 4.3 Fabrication of Quartz/Cu_2_AgBiI_6_/CuI

To fabricate Quartz/Cu_2_AgBiI_6_/CuI thin films, Cu_2_AgBiI_6_ was deposited on quartz as described above. Copper­(I) iodide (Alpha Aesar Puratronic, 99.998%) was subsequently deposited in the same chamber, using the same CuI source, and during the same deposition. To do this, after Cu_2_AgBiI_6_ was deposited, the substrate shutter was closed, and the BiI_3_ and AgI sources were cooled to room temperature. The temperature of the CuI source was initially held at approximately 100 °C (corresponding to a CuI evaporation rate of 0 Å s^–1^) until the chamber pressure decreased to 10^–6^ mbar. Subsequently, the temperature was increased, and once a stable CuI deposition rate of 0.5 Å s^–1^ was achieved, the shutter was opened, and a nominal CuI thickness of 200 nm was deposited on the Cu_2_AgBiI_6_ film. Films were not annealed postdeposition.

### 4.4 Fabrication of Quartz/PCBM/Cu_2_AgBiI_6_

To fabricate quartz/PCBM/Cu_2_AgBiI_6_ samples, [6,6]-phenyl-C61-butyric acid methyl ester (PCBM) was prepared by dissolving 20 mg/mL in a 3:1 chlorobenzene/dichlorobenzene mixture and stirred for 15 min at room temperature inside a N_2_ glovebox (O_2_ and H_2_O < 5 ppm). Prior to deposition, the solution was filtered using a 0.22 μm PTFE filter (13 mm diameter, Gilson Scientific Ltd.). The PCBM solution was then deposited on the quartz substrates inside an N_2_ glovebox (O_2_ and H_2_O < 5 ppm) by dynamically spin coating 50 μL at 2000 rpm for 30 s followed by annealing at 100 °C for 1 min. After the deposition of PCBM, the samples were transferred to the evaporation chamber and Cu_2_AgBiI_6_ was deposited as described above. The samples were not annealed postdeposition.

### 4.5 Fabrication of Quartz/SnO_2_/Cu_2_AgBiI_6_

SnO_2_ was deposited onto z-cut quartz substrates by first following the same substrate cleaning procedure as previously described for quartz/Cu_2_AgBiI_6_ films, with the exception that the substrates underwent UV-ozone cleaning for 30 min instead of 15 min. Upon finishing the cleaning procedure, planar SnO_2_ was immediately deposited by spin coating 200 μL of a 2% mixture of SnO_2_ nanoparticles (Fisher Scientific, 15% colloidal dispersion in H20) in ultrapure water (Cayman Chemical, item no. 400000) in air at 4000 rpm spin speed, 2000 rpms^–1^ ramp rate, for 30 s, followed by immediately annealing in air at 150 °C for 30 min before quenching to room temperature. Cu_2_AgBiI_6_ was then fabricated as described above. Films were not annealed postdeposition.

### 4.6 Absorption Spectroscopy

Reflectance (*R*) and transmittance (*T*) spectra were measured using a Fourier transform infrared (FTIR) spectrometer (Bruker VERTEX 80v), configured with a tungsten halogen lamp illumination source, a CaF_2_ beamsplitter and a silicon diode detector. Absorbance (*A*) is calculated with the following equation

2 $$A=-\log_{10}\left(\frac{T}{1-R}\right)$$

The absorption coefficient (α) spectra were calculated by dividing A by the thickness obtained from CABI as shown in Table S1 of the Supporting Information.

### 4.7 X-ray Diffraction

The X-ray diffraction (XRD) patterns were measured in air using a Panalytical Empyrean powder diffractometer with a copper X-ray source (Cu Kα_1_ X-rays with a wavelength of 1.5406 Å). The scan range was from 10.0° to 45.0° and the scan step size was set as 0.004°. The raw XRD patterns were then corrected for tilt by shifting the 2θ-axis to the z-cut quartz reference peak, which is at 2θ = 16.433°.^69^ The Pawley fitting implemented in the Highscore Plus software was used to assign unit cells (space group and lattice parameters) of the known phases to the measured XRD patterns. For Q/SnO_2_/CABI, the XRD pattern was obtained with ten times longer acquisition time to achieve a comparable signal-to-noise ratio.

### 4.8 Scanning Electron Microscopy

Top-down and cross-sectional SEM images were taken on a FEI Quanta 600 FEG at 5 kV acceleration voltage with current defined by spot size 3.0.

### 4.9 Optical-Pump Terahertz-Probe Spectroscopy

Our optical-pump terahertz-probe spectroscopy (OPTPS) setup uses a Spectra Physics Mai Tai-Ascend-Spitfire Pro Ti/sapphire regenerative amplifier. The amplifier generates ultrafast laser pulses with 35 fs pulse duration, 800 nm center wavelength and 5 kHz repetition rate. An optical pump excitation wavelength of 530 nm was achieved by using a traveling-wave optical parametric amplifier of superfluorescence (TOPAS) with a sum-frequency generation of the signal pulse. THz probe pulses were generated with a spintronic emitter which consists of 1.9 nm Tungsten/2.0 nm Co_40_Fe_40_Be_20_/1.9 nm platinum coated with antireflectivity and high-reflectivity coatings.^70^ The sample stored in an evacuated chamber at pressure less than 10^–1^ mbar was excited with the pump and probed with the THz pulse shortly after. The pump and THz pulses were chopped with optical choppers with frequencies of 1.25 and 2.5 kHz respectively to obtain the THz transmission change Δ*T*. The sigma of pump and probe beam at the sample positions were measured as 1.75 mm and 0.33 mm, respectively. The power of the pump pulses was tuned by an ND filter wheel. The THz transmission from thin films was detected by electro-optic sampling in a (110)-ZnTe crystal (1 mm thickness) with a spatially and temporally overlapping 800 nm gate pulse. The THz transmission was measured at the peak of the THz pulse for different delays of the pump beam, mapping the THz transmission as a function of time after photoexcitation. The OPTPS decay traces with different delays were measured for 3 fluences on both quartz and film side.

## Supplementary Material



## Notes

Additional details on FTIR, SEM, XRD, and OPTPS analysis (PDF)
