# Supplementary material for: Impact of Charge Transport Layers on the Structural and Optoelectronic Properties of Coevaporated Cu2AgBiI6
Source: ACS Appl Mater Interfaces. 2025 Jul 8;17(28):40363–74. doi: 10.1021/acsami.5c05243 (PMC12278222; doi:10.1021/acsami.5c05243)
Supplement: Supplementary file 1 [file am5c05243_si_001.pdf]

Supporting Information

Impact of Charge Transport Layers on the Structural and Optoelectronic Properties of Coevaporated Cu_2AgBiI_6

Jae Eun Lee[a], Marcello Righetto[a], Benjamin W. J. Putland[a], Siyu Yan[a], Joshua R. S. Lilly[a], Snigdha Lal[a], Heon Jin[a], Nakita K. Noel[a], Michael B. Johnston[a], Henry J. Snaith[a], and Laura M. Herz[a]*

[a] Department of Physics, Clarendon Laboratory, University of Oxford, Parks Road, Oxford, OX1 3PU, UK

* Corresponding author (*laura.herz@physics.ox.ac.uk*)

Table of Contents

S1. Scanning Electron Microscopy Images ***3***

S2. Band Gap Extraction Methods ***5***

S2.1 Elliott Fitting **5**

S2.2 Point of Inflection Method **6**

S2.3 Tauc Method **6**

S3. Pawley Fits ***12***

S4. OPTPS Transient Analysis ***18***

S4.1. Charge-carrier Localization in Cu_2AgBiI_6 and Two-level Mobility Model **18**

S4.2 Charge-carrier Recombination After Ultrafast Localization Process **22**

S4.3. Instrument Response of OPTPS **27**

References ***28***

S1. Scanning Electron Microscopy Images

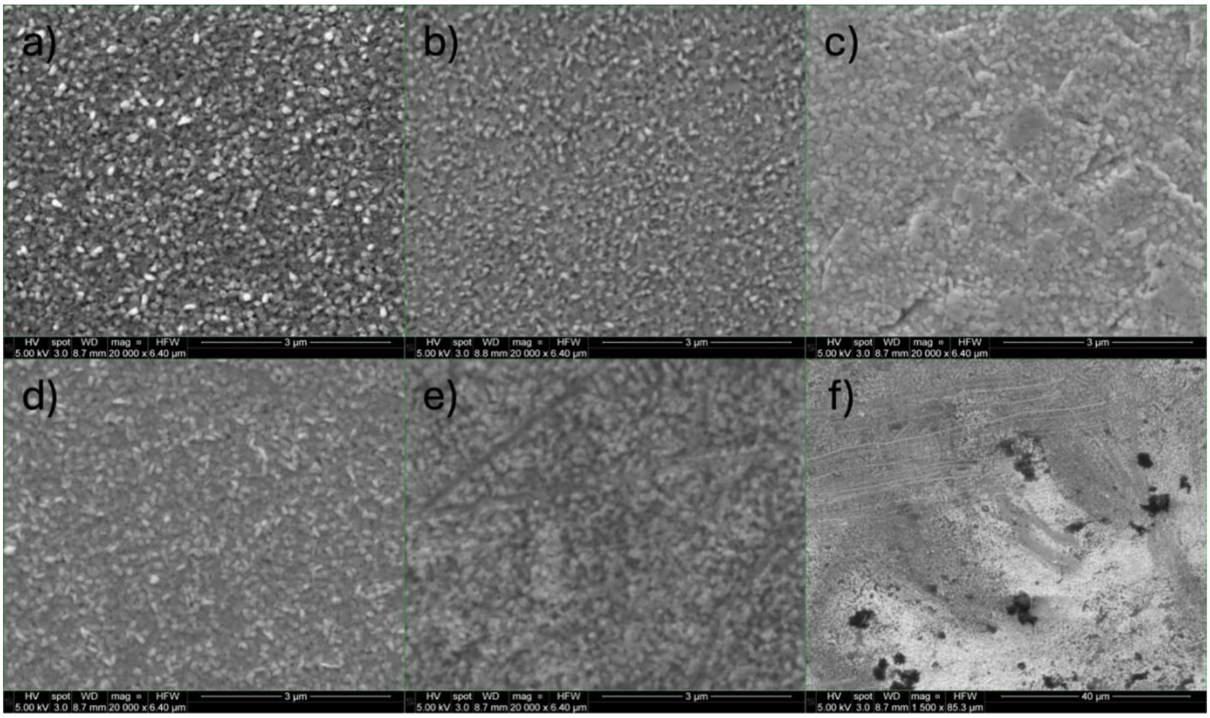

Figure S1 Top-view SEM images of (a) Q/CABI, (b) Q/CABI/PTAA, (c) Q/CABI/CuI, (d) Q/PCBM/CABI, (e) Q/SnO_2/CABI, and (f) Q/SnO_2/CABI with lower magnification

The top-down SEM images of Q/CABI reveal small grains and bright spots, which may originate from small BiI_3 impurities evidenced from the XRD pattern. Although an assessment of the Q/CABI/PTAA and Q/CABI/CuI top-down SEMs is ambiguous owing to the deposition of PTAA and CuI on top of CABI, more uniform SEM images of Q/CABI/PTAA suggests uniform deposition of PTAA. However, the Q/CABI/CuI SEM image shows a non-uniform surface, which is in line with the XRD and OPTPS analysis that CABI reacts with CuI to form a more Cu-rich interface. The SEM image of Q/SnO_2/CABI is non-uniform with distinct bright and dark regions. This is also in line with the XRD and OPTPS analysis suggesting that CABI is not properly grown on SnO_2, with the incomplete reaction of the binary precursors and formation of additional trigonal phases leading to poor morphology.

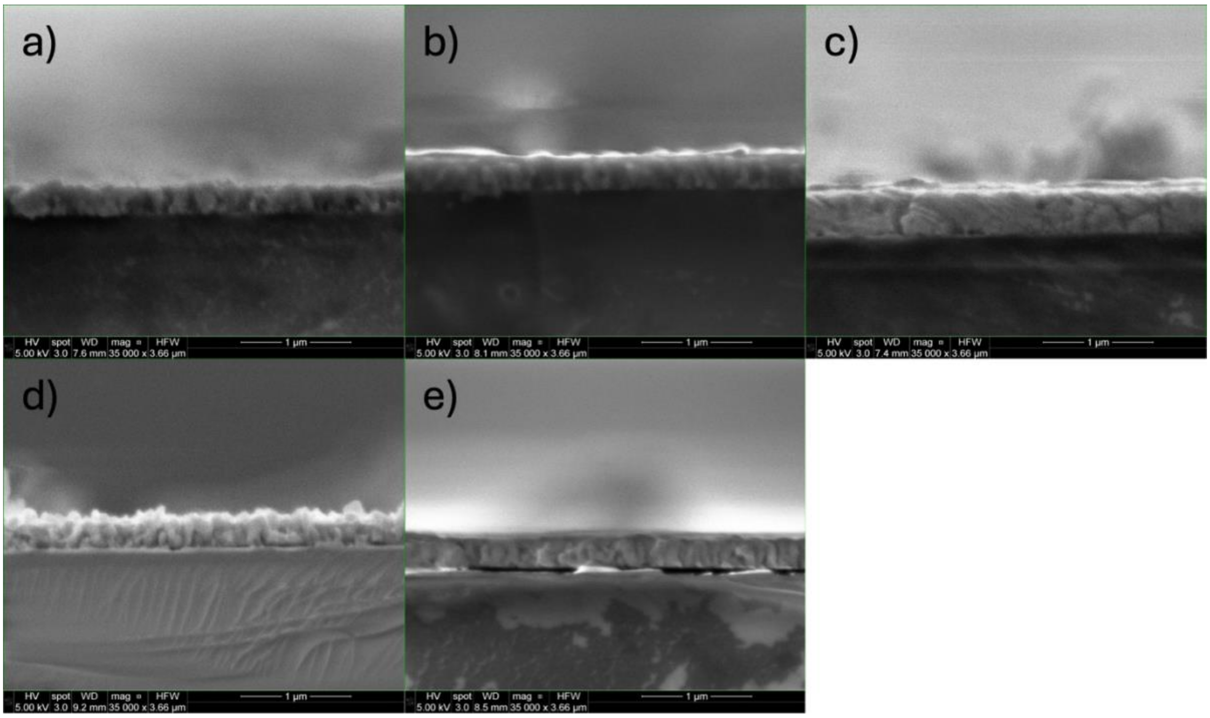

Figure S2 Cross-sectional SEM images of (a) Q/CABI, (b) Q/CABI/PTAA, (c) Q/CABI/CuI, (d) Q/PCBM/CABI, (e) Q/SnO_2/CABI

Table S1 Thicknesses of CABI films determined from cross-sectional SEM, including both CABI and, where present, charge transport layers.

Sample	Thickness (nm)
Q/CABI	240
Q/CABI/PTAA	288
Q/CABI/CuI	416
Q/PCBM/CABI	256
Q/SnO2/CABI	262

S2. Band Gap Extraction Methods

S2.1 Elliott Fitting

The absorption coefficient spectra were fitted by the Elliott model to obtain the optical bandgap (E_{g_Elliot}), exciton binding energy (E_b) and absorption edge broadening parameter (γ_{Abs})[1]. The Elliott model represents the total absorption coefficient, $\alpha(E)$ as a linear combination of the absorption coefficient from bound excitons, $\alpha_{EX}(E)$, and electron-hole continuum states, $\alpha_C(E)$, for a direct semiconductor as shown below[1-3]:

$$\alpha(E) = \alpha_{EX}(E) + \alpha_C(E) \tag{S1}$$

The contribution from $\alpha_{EX}(E)$ is expressed as:

$$\alpha_{EX}(E) = \frac{b_0}{E}\sum_{n=1}^{\infty}\frac{4\pi E_b^{3/2}}{n^3}\delta\left(E - \left[E_{g\_Elliott} - \frac{E_b}{n^2}\right]\right) \tag{S2}$$

where b_0 is a constant of proportionality that includes the electric dipole transition matrix element between the valence and conduction band, $|\langle\Psi_c|P|\Psi_v\rangle|^2$, and n is a positive integer quantum number. Equation S4 describes line series at energies $-E_b/n^2$ below $E_{g\_Elliott}$ and the magnitudes are proportional to $1/n^3$.

The contribution from $\alpha_C(E)$ is expressed as:

$$\alpha_C(E) = \frac{b_0}{E}\left[\frac{2\pi\sqrt{\frac{E_b}{E - E_{g\_Elliot}}}}{1 - \exp\left(-2\pi\sqrt{\frac{E_b}{E - E_{g\_Elliot}}}\right)}\right]c_0^{-1}JDoS(E) \tag{S3}$$

where the joint density of states, $JDoS$ is given by :

$$JDoS(E) = \begin{cases} c_0\sqrt{E - E_{g\_Elliot}}, & for\ E > E_{g\_Elliot} \\ 0, & otherwise \end{cases} \tag{S4}$$

and the joint density of states constant (c_0) is given by:

$$c_0 = \frac{1}{(2\pi)^2}\left(\frac{2\mu}{\hbar^2}\right)^{3/2} \times 2 \tag{S5}$$

where μ is the reduced effective mass of the electron-hole system, which is assumed to be 0.375 m_e[4]. The term in the square brackets in Equation S3 is the Coulombic enhancement factor, which represents the probability of an electron and a hole existing in the same space. The second term $c_0^{-1}JDoS(E)$ represents the absorption coefficient of free electrons and holes in the absence of Coulombic attraction, $\alpha_{Free}(E)$.

The linear combination of the contributions from the excitonic and continuum states as shown in Equation S1 is then convolved with a broadening function, which is written as:

$$g(E) = \frac{1}{\cosh \frac{E - E_b}{\gamma_{Abs}}} \quad \text{(S6)}$$

where γ_{Abs} is the absorption edge broadening parameter.

S2.2 Point of Inflection Method

The optical bandgaps were alternatively obtained by using the point of inflection method, which estimates the bandgap from the steepest gradient of the absorption onset. This method has been previous used by Savill et al. and Lal et al. to calculate bandgaps from weak and broadened absorption onset, where Elliott fitting can be impractical[5, 6]. To obtain the steepest gradient, the raw absorption onset was fitted with an arbitrary polynomial function and differentiated using numpy.diff. The peak of the first derivative is taken as the optical band gap (E_{g_inf}).

S2.3 Tauc Method

For a direct band gap semiconductor, the optical bandgap (E_{g_Tauc}) can be estimated by the following equation:

$$\alpha(E)hv \propto \left(hv - E_{g\_Tauc}\right)^{1/2} \quad \text{(S7)}$$

To improve the accuracy of the Tauc method, especially for thin films interfaced with charge transport layers, we have fitted a linear baseline in the sub-band gap region. The extracted Tauc bandgap value (E_{g_Tauc}) is the energy where the baseline fit and the linear fit to the square of the absorption coefficient onset meet.

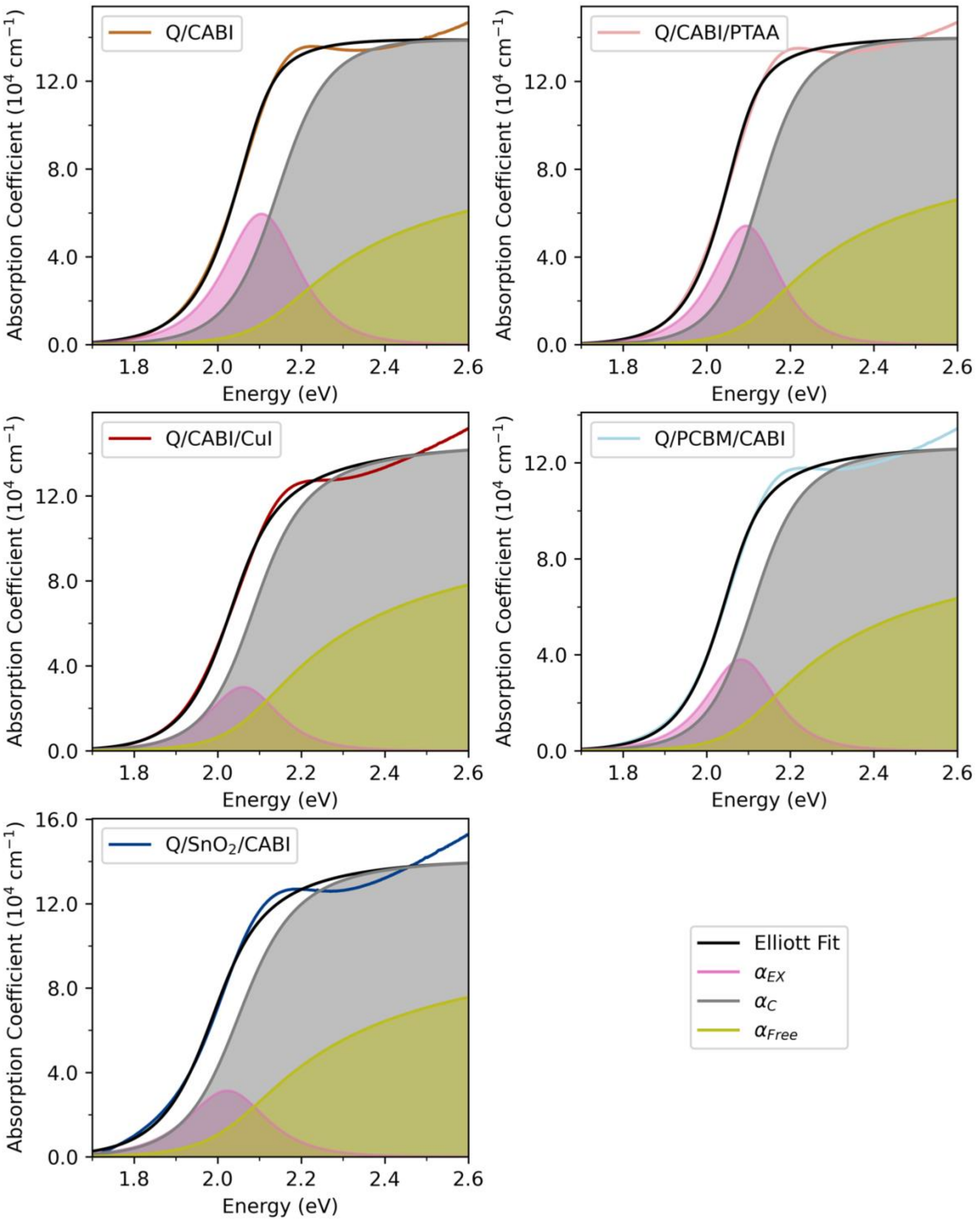

Figure S3 Absorption coefficient spectra of CABI thin film on quartz and CABI thin films with transport layers on quartz, shown near the absorption onset together with the Elliott fit. α_{EX}, α_{C} and α_{Free} represent the absorption coefficient contributions from bound excitons, electron-hole continuum states and free electron and holes respectively.

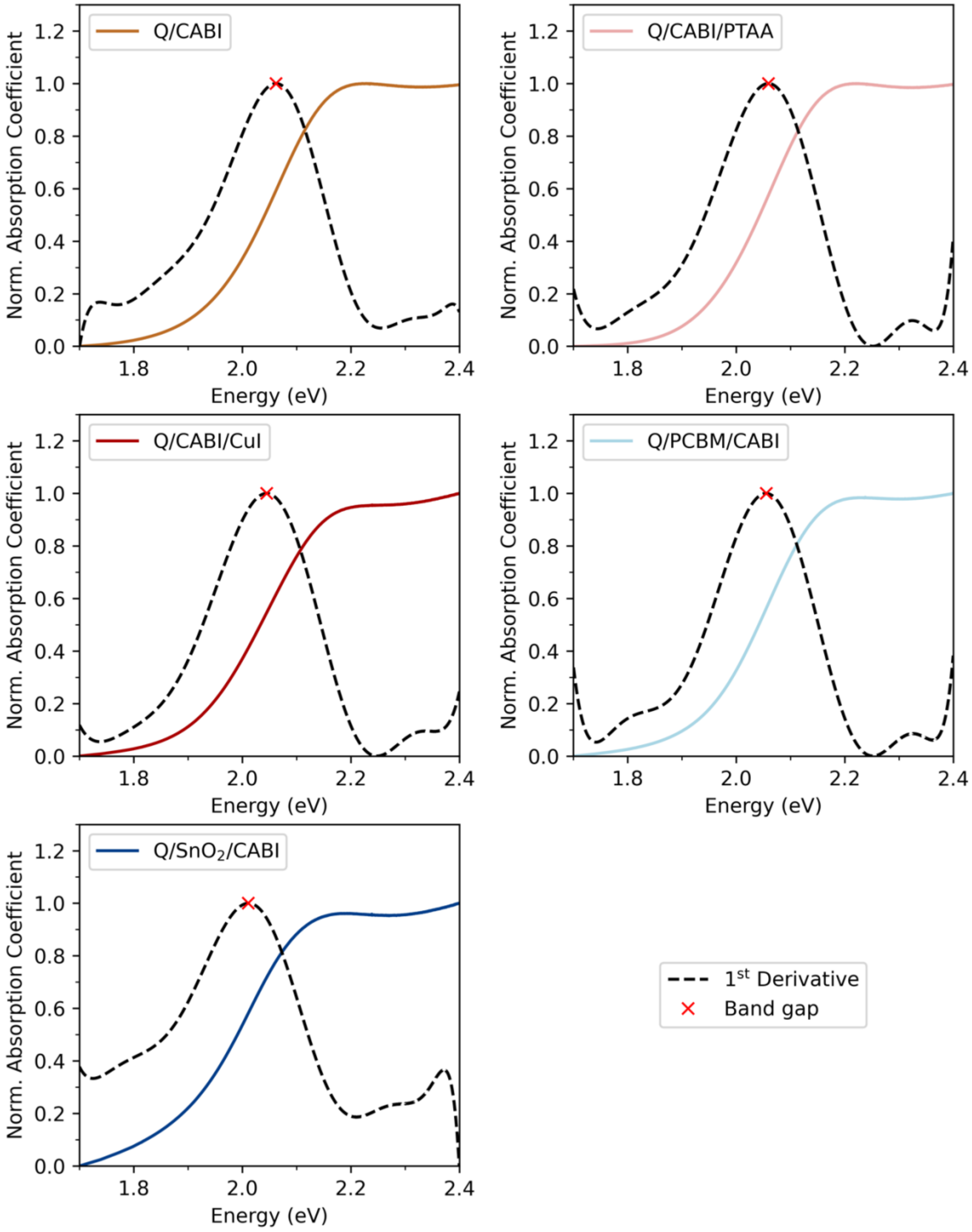

Figure S4 Normalised absorption coefficient spectra of CABI thin film on quartz and CABI thin films with transport layers on quartz, shown near the absorption onset together with their first derivatives. The red cross indicates the maximum of the first derivative, which corresponds to the extracted band gap value.

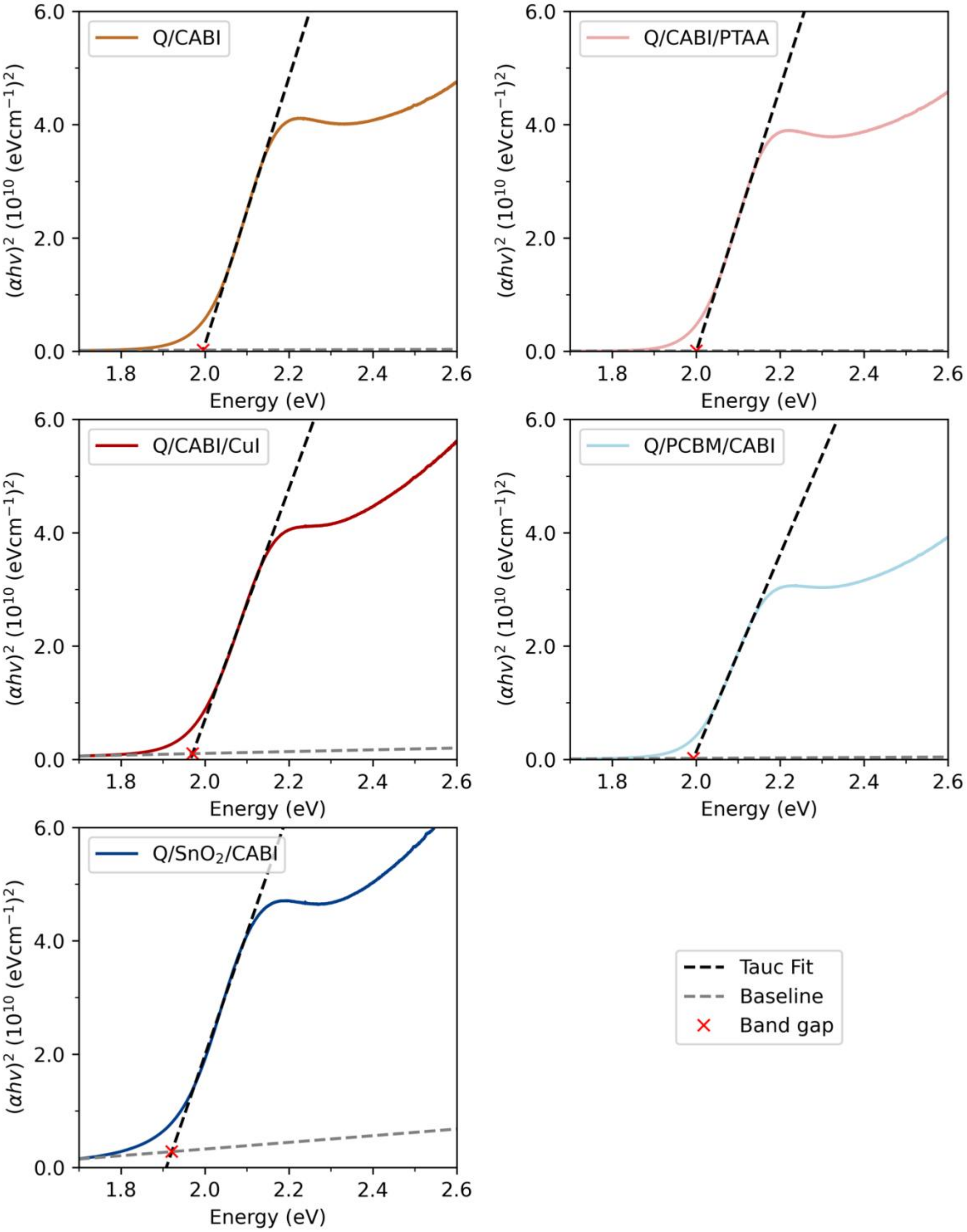

Figure S5 Tauc plot of CABI thin film on quartz and CABI thin films with transport layers on quartz. The red cross indicates the point corresponding to the extracted bandgaps, i.e. the energy where the baseline fit and the linear fit to the square of the absorption coefficient onset meet.

Table S2 Summary of the extracted absorption parameters of CABI thin film and CABI thin films with transport layers, all on quartz substrates.

Sample	Elliott Fit Parameters			E_{g_inf} (eV)	E_{g_Tauc} (eV)
	E_{g_Elliot} (eV)	E_b (eV)	γ_{Abs} (eV)		
Q/CABI	2.13	0.036	0.076	2.06	2.00
Q/CABI/PTAA	2.12	0.031	0.069	2.06	2.00
Q/CABI/CuI	2.06	0.016	0.073	2.05	1.97
Q/PCBM/CABI	2.10	0.026	0.072	2.06	1.99
Q/SnO_2/CABI	2.01	0.016	0.082	2.01	1.92

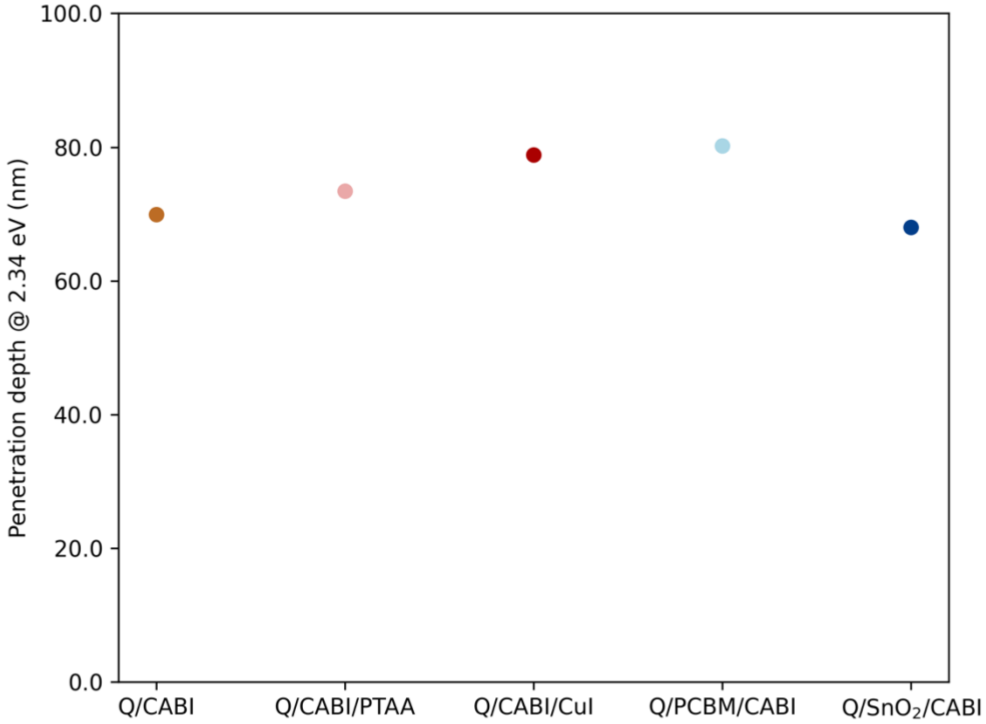

Figure S6 Penetration depth of light incident on CABI films at the excitation energy of 2.34 eV, calculated as the inverse of the absorption coefficient at 2.34 eV.

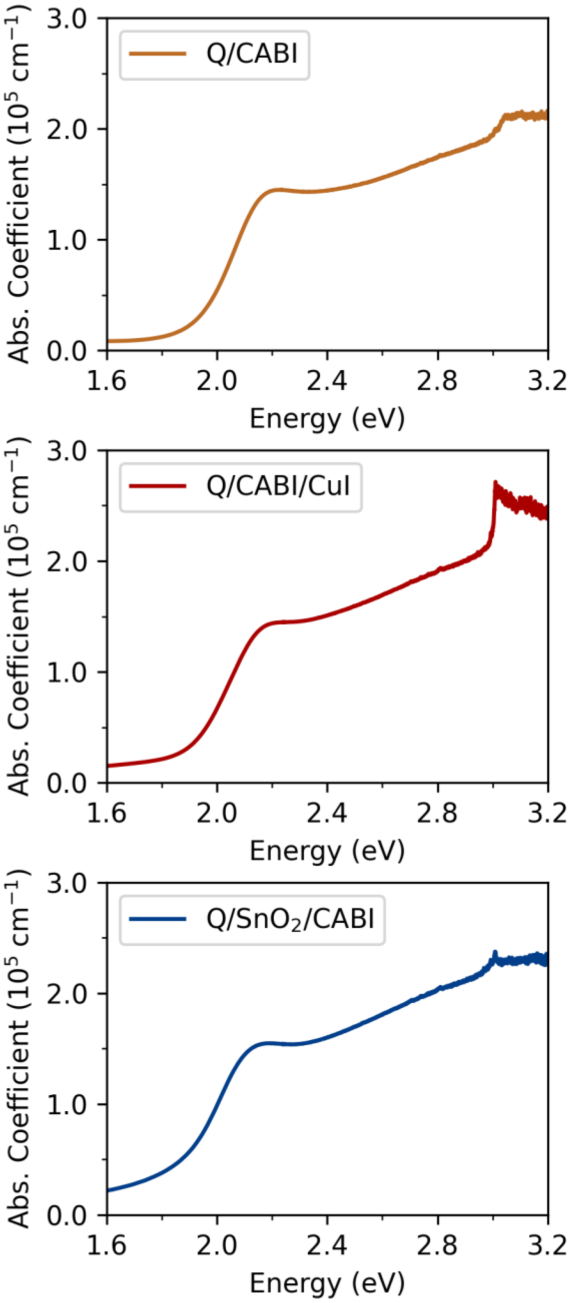

Figure S7 Absorption coefficient spectra of the CABI thin films with and without charge transport layers, on quartz substrates, covering a wider range of photon energies.

S3. Pawley Fits

Table S3 The structural and phase properties of the Cu_2AgBiI_6 phase for the thin films obtained through Pawley fitting to the XRD data as shown in Figures S8 - S12. The Bragg reflection of the quartz substrate was used as the internal standard to compare the lattice parameters. The brackets next to data represent uncertainty in the Pawley fitting calculation. For c/2a and volume, the errors were propagated from the unit cell.

Thin Films	Cu_2AgBiI_6 Phase				Impurities	GoF [1]
	Space Group	Unit Cell (Å)	c/2a	Volume (Å^3)		
Q/CABI	$R\bar{3}m$	a/b = 4.3167(3)<br>c=20.797(2)	2.4088(3)	335.61(5)	BiI_3	1.09
Q/CABI/PTAA	$R\bar{3}m$	a/b= 4.3135(3)<br>c=20.751(2)	2.4054(3)	334.38(4)	BiI_3	0.95
Q/CABI/CuI	$R\bar{3}m$	a/b= 4.308(1)<br>c=20.723(5)	2.4052(8)	333.1(1)	CuI, BiI_3, Trigonal Ternary phases	1.01
Q/PCBM/CABI	$R\bar{3}m$	a/b= 4.332(1)<br>c=20.747(3)	2.3947(8)	337.2(2)	BiI_3	0.94
Q/SnO_2/CABI	$R\bar{3}m$	a/b= 4.328(1)<br>c=20.757(1)	2.3982(8)	336.7(2)	AgI, CuI, BiI_3, Trigonal Ternary phases	1.21

[1] goodness of fit

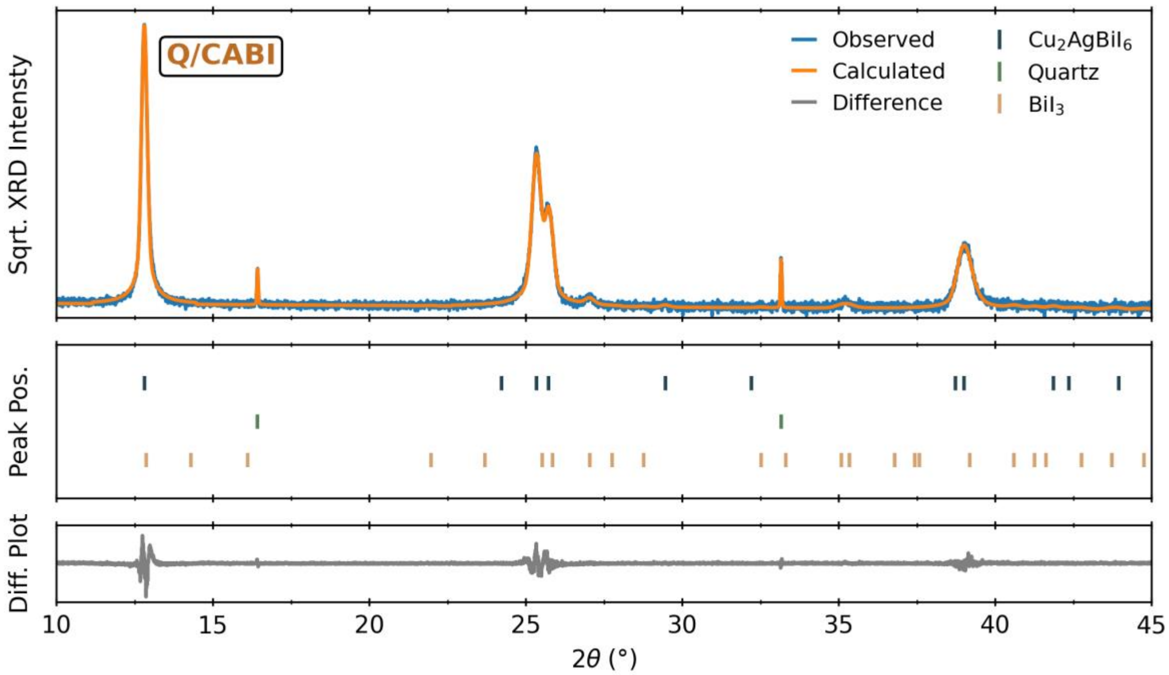

Figure S8 Pawley fits to the XRD patterns for the Quartz/CABI sample. The calculated pattern is overlaid on the experimental pattern in the upper panel and the difference between the two is shown in the bottom panel in grey. The contribution to the calculated pattern from each phase is shown with vertical markers in the middle panel. The XRD intensity is presented on a square root scale.

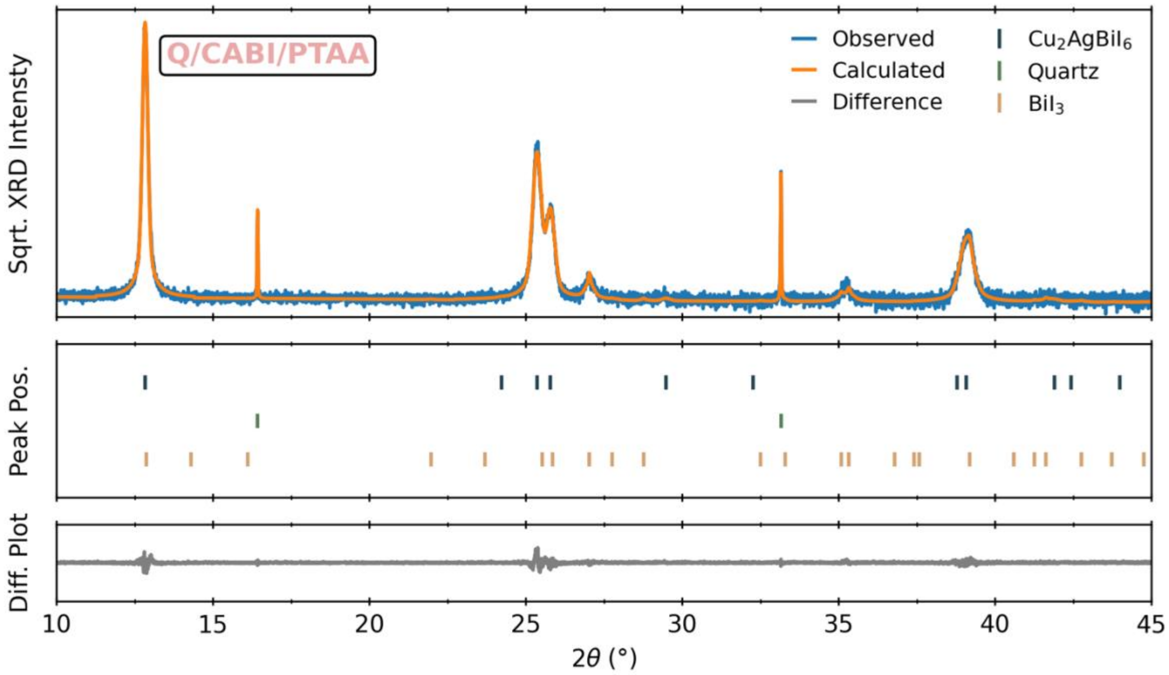

Figure S9 Pawley fits to the XRD patterns for the Quartz/CABI/PTAA sample. The calculated pattern is overlaid on the experimental pattern in the upper panel and the difference between the two is shown in the bottom panel in grey. The contribution to the calculated pattern from each phase is shown with vertical markers in the middle panel. The XRD intensity is presented on a square root scale.

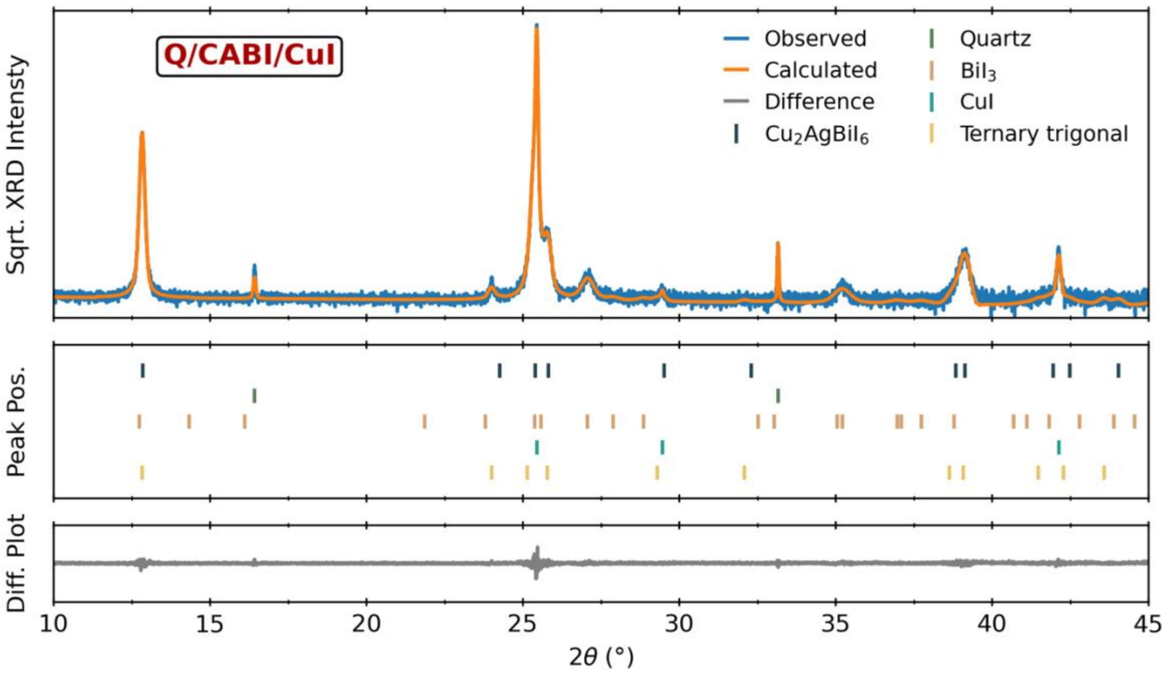

Figure S10 Pawley fits to the XRD patterns for the Quartz/CABI/CuI sample. The calculated pattern is overlaid on the experimental pattern in the upper panel and the difference between the two is shown in the bottom panel in grey. The contribution to the calculated pattern from each phase is shown with vertical markers in the middle panel. The XRD intensity is presented on a square root scale.

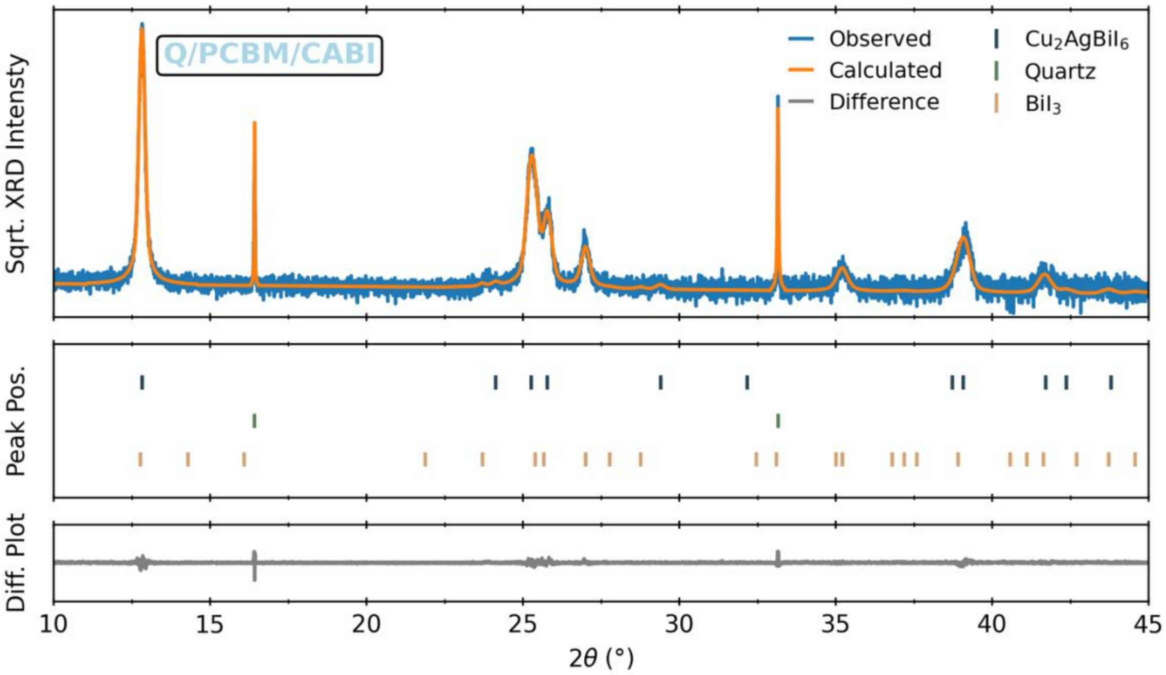

Figure S11 Pawley fits to the XRD patterns for the Quartz/PCBM/CABI sample. The calculated pattern is overlaid on the experimental pattern in the upper panel and the difference between the two is shown in the bottom panel in grey. The contribution to the calculated pattern from each phase is shown with vertical markers in the middle panel. The XRD intensity is presented on a square root scale.

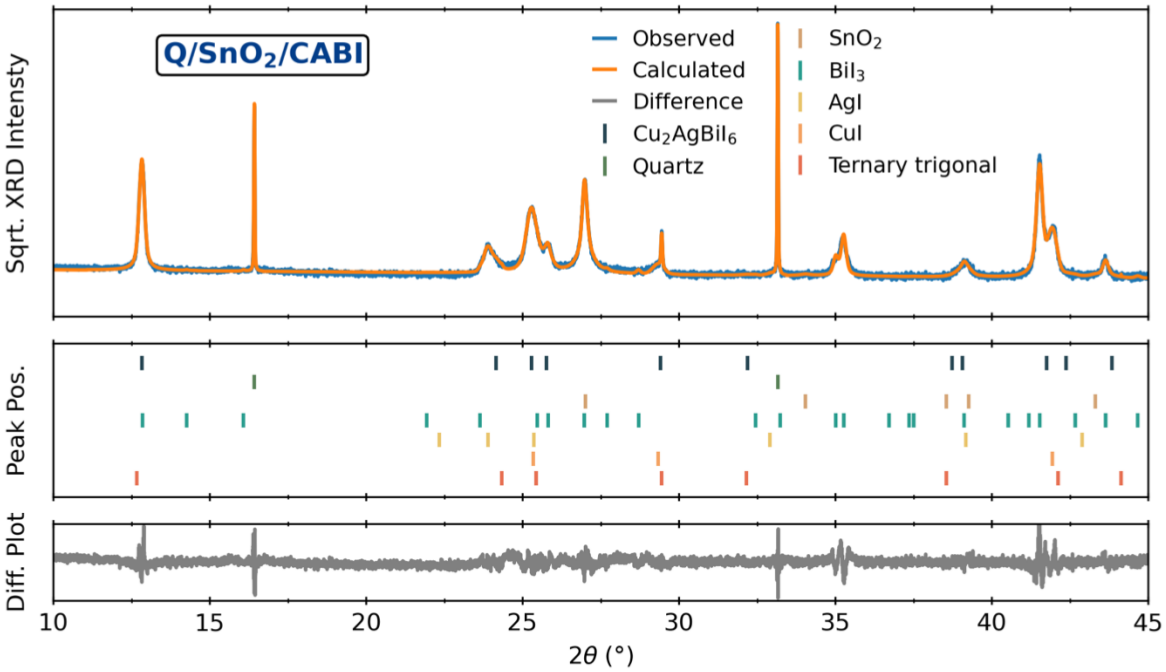

Figure S12 Pawley fits to the XRD patterns for the Quartz/SnO_2/CABI sample. The calculated pattern is overlaid on the experimental pattern in the upper panel and the difference between the two is shown in the bottom panel in grey. The contribution to the calculated pattern from each phase is shown with vertical markers in the middle panel. The XRD intensity is presented on a square root scale.

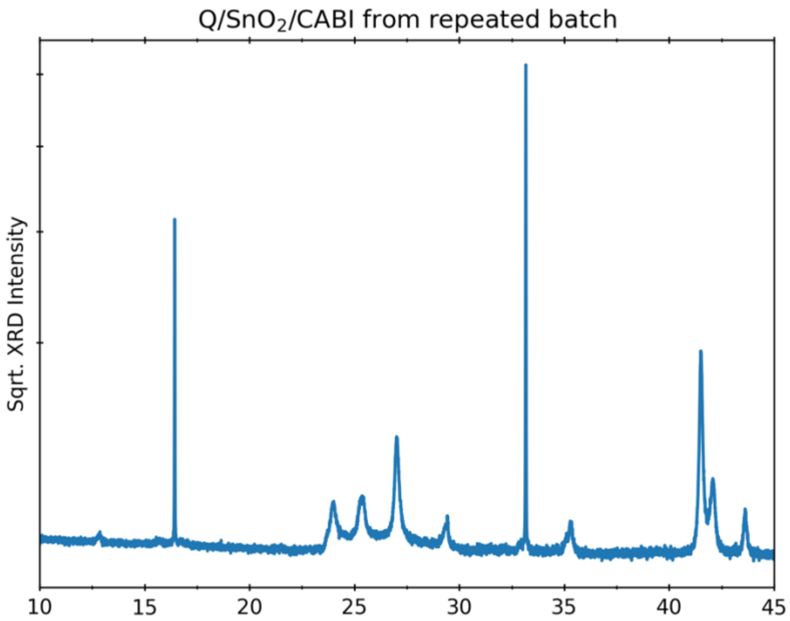

Figure S13 XRD pattern of a Q/SnO_2/CABI sample from a repeated coevaporated batch.

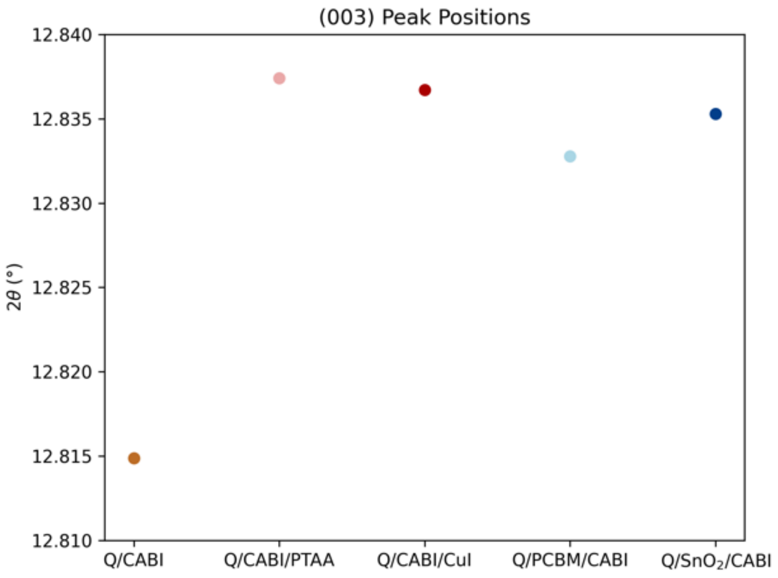

Figure S14 Extracted peak positions in XRD spectra for the (003) diffraction peak of CABI films and CABI half stacks with charge transport layers, on quartz.

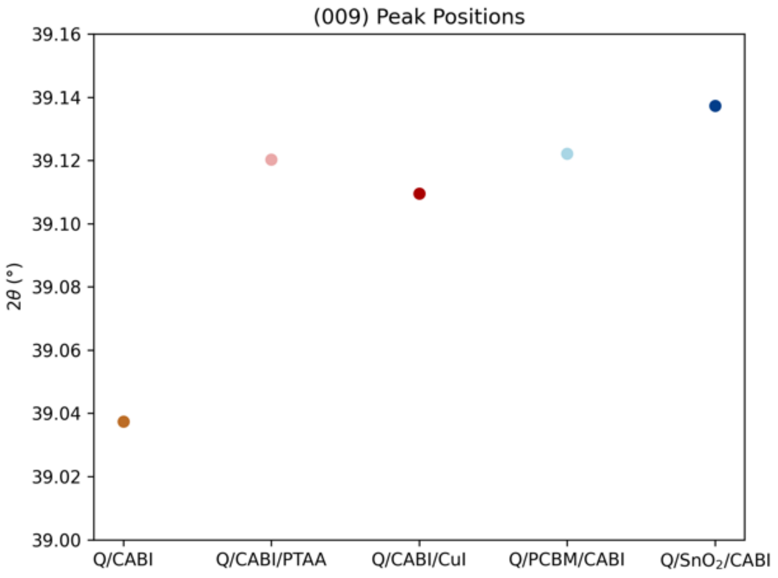

Figure S15 Extracted peak positions in XRD spectra for the (009) diffraction peak of CABI films and CABI half stacks with charge transport layers, on quartz.

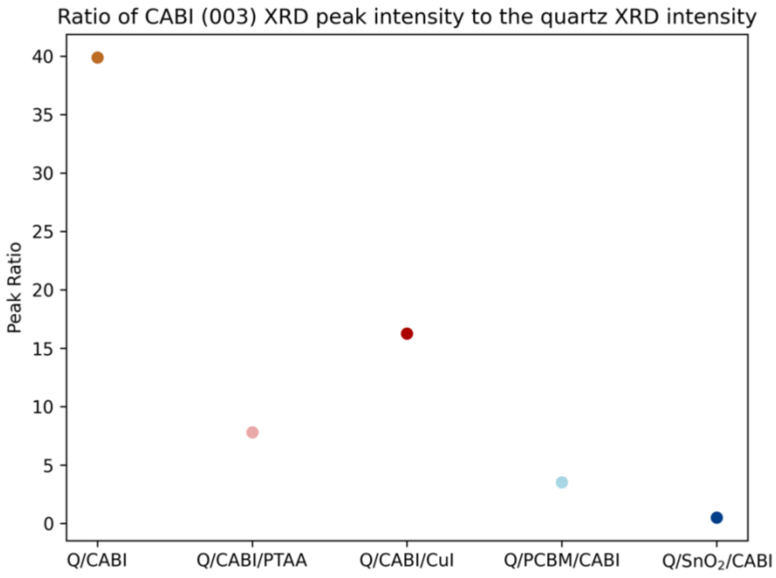

Figure S16 The peak ratio of the CABI (003) XRD peak intensity to the quartz XRD peak intensity.

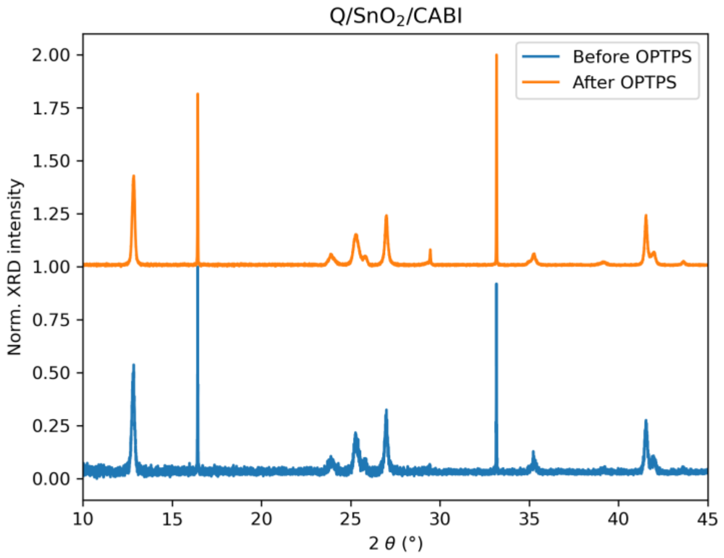

Figure S17 XRD patterns of $Q/SnO_2/CABI$ before and after an OPTPS measurement.

This indicates that $Q/SnO_2/CABI$ remains structurally stable under pulsed illumination and vacuum conditions, despite the presence of additional impurity phases.

S4. OPTPS Transient Analysis

S4.1. Charge-carrier Localization in Cu_2AgBiI_6 and Two-level Mobility Model

Charge-carrier localization has been reported for a range of semiconductors in the CuI-AgI-BiI_3 phase space such as Cu_2AgBiI_6,[4, 7] $Cu_{4x}(AgBi)_{1-x}I_4$[7] and $(AgI)_x(BiI_3)_y$[5]. These materials exhibit an ultrafast decay of the initial THz photoconductivity within a few picoseconds, followed by a slow subsequent decay. Although the ultrafast early decay could originate from other processes such as charge-carrier cooling, exciton formation or charge-carrier trapping, the fluence-independent THz photoconductivity transients and temperature-dependent optical-pumped THz-probe spectroscopy (OPTPS) experiments strongly supported a charge-carrier localization behaviour[4, 7, 8]. In particular, temperature-dependent OPTPS on Cu_2AgBiI_6 has revealed an opposite behaviour for the initial and later OPTPS signals[7]. The initial OPTPS signal increased with decreasing temperatures, reflecting bandlike transport associated with large polarons, whereas the later OPTPS signal decreases with decreasing temperatures, showing temperature-activated "hopping" transport of a localised state or small polaron.

To describe the charge-carrier localization process in Cu_2AgBiI_6, Buizza et al. have proposed a two-level mobility model to describe the OPTPS photoconductivity transients[7]. In this model, the photoconductivity of the material originates from two different states with different populations and mobilities. An initial delocalised state (N_{deloc}, μ_{deloc}) is populated immediately after the photoexcitation but these charge carriers rapidly transfer to a localised state (N_{loc}, μ_{loc}) as a result of the charge-carrier localization process. Therefore, the resulting sheet photoconductivity (ΔS) can be expressed as

$$\Delta S = \frac{e}{A_{eff}}(N_{deloc}\mu_{deloc} + N_{loc}\mu_{loc}) \qquad \text{(S8)}$$

where A_{eff} is the effective area of the overlap of optical pump and THz probe pulse considering the Gaussian beam profile. As reported by Xia et al., A_{eff} is defined as $A_{eff} = 2\pi(\sigma_{pump}^2 + \sigma_{THz}^2)$, where σ_{pump} and σ_{THz} are the pump and THz beam waists, respectively[9]. To avoid artifacts, the ratio between σ_{pump} and σ_{THz} is fixed at ~ 6[10].

By assuming a predominantly monomolecular charge-carrier recombination regime in the low fluence range, we can describe the charge-carrier population of the two states by the following set of coupled rate equations:

$$\begin{cases} \frac{dN_{deloc}}{dt} = -k_{loc}N_{deloc}(t) \\ \frac{dN_{loc}}{dt} = k_{loc}N_{deloc}(t) - k_R N_{loc}(t) \end{cases} \qquad \text{(S9)}$$

where k_{loc} and k_R are the charge-carrier localization and average recombination rates, respectively. The analytical solution to this set of coupled rate equations yields the time-dependent charge-carrier population for the two states. As described previously, the resulting $\Delta T/T$ signal is obtained by combining Equation S8 and S9.

$$\frac{\Delta T}{T} = -\frac{eN}{\epsilon_0 c(n_A + n_B)A_{eff}}\left(\left(\mu_{deloc} - \frac{\mu_{loc}k_{loc}}{k_{loc} - k_R}\right)e^{-k_{loc}t} + \frac{\mu_{loc}k_{loc}}{k_{loc} - k_R}e^{-k_R t}\right) \quad \text{(S10)}$$

where n_A and n_B are the refractive indices of the optically thick (at THz frequencies) materials interfacing the films, i.e. quartz and air, respectively. For the quartz side, n_A is set as 2.13[11]. For the film/air side, n_B is set as 1.

To account for the instrument response function of the OPTPS system used here, Equation S10 was convolved with a normalised Gaussian pulse with σ_{IRF} = 0.34 ps (Refer to Figure S23). Accounting for the time zero offset t_0, the overall analytical expression used to fit the OPTPS data was derived as:

$$\frac{\Delta T}{T} = -\frac{eN}{2\epsilon_0 c(n_A + n_B)A_{eff}}\left(\left(\mu_{deloc} - \frac{\mu_{loc}k_{loc}}{k_{loc} - k_R}\right)e^{-k_{loc}(t-t_0)+\frac{k_{loc}^2\sigma_{IRF}^2}{2}}Erfc\left(\frac{k_{loc}\sigma_{IRF}^2 - (t-t_0)}{\sqrt{2}\sigma_{IRF}}\right) + \frac{\mu_{loc}k_{loc}}{k_{loc} - k_R}e^{-k_R(t-t_0)+\frac{k_R^2\sigma_{IRF}^2}{2}}Erfc\left(\frac{k_R\sigma_{IRF}^2 - (t-t_0)}{\sqrt{2}\sigma_{IRF}}\right)\right) \quad \text{(S11)}$$

Here, $Erfc(x)$ is the complementary error function, defined as:

$$Erfc(x) = \frac{2}{\sqrt{\pi}}\int_x^\infty e^{-t^2}dt \quad \text{(S12)}$$

The OPTP transients were globally fitted across all three fluences up to 10 ps, yielding values for μ_{loc}, μ_{deloc}, k_{loc}. k_R was fixed to the value obtained from long-time OPTPS fitting to obtain more accurate values for the early time parameters.

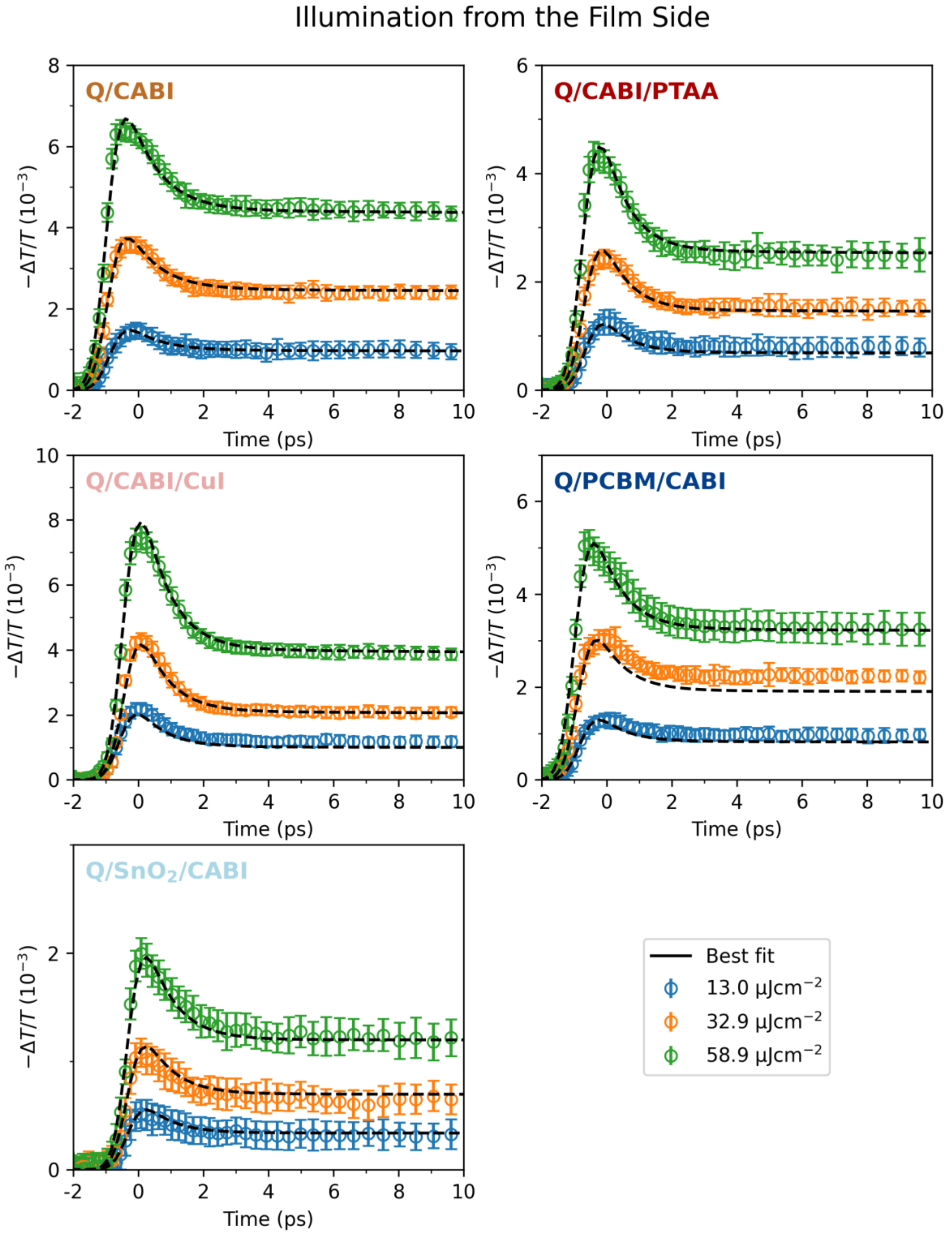

Figure S18 OPTPS photoconductivity transients shown for times between -2 and 10 ps after excitation for Cu_2AgBiI_6 thin films and half stacks with hole transport layers (PTAA and CuI) and electron transport layers (PCBM and SnO_2), deposited on a quartz substrate. Samples were illuminated from the film side (i.e. the side opposite the quartz substrate) with excitation pump fluences of 13.0, 32.9 and 58.9 μJcm^{-2}. The dotted black lines are global fits to the fluence-dependent OPTPS transients using the localization model described in Section S4.1.

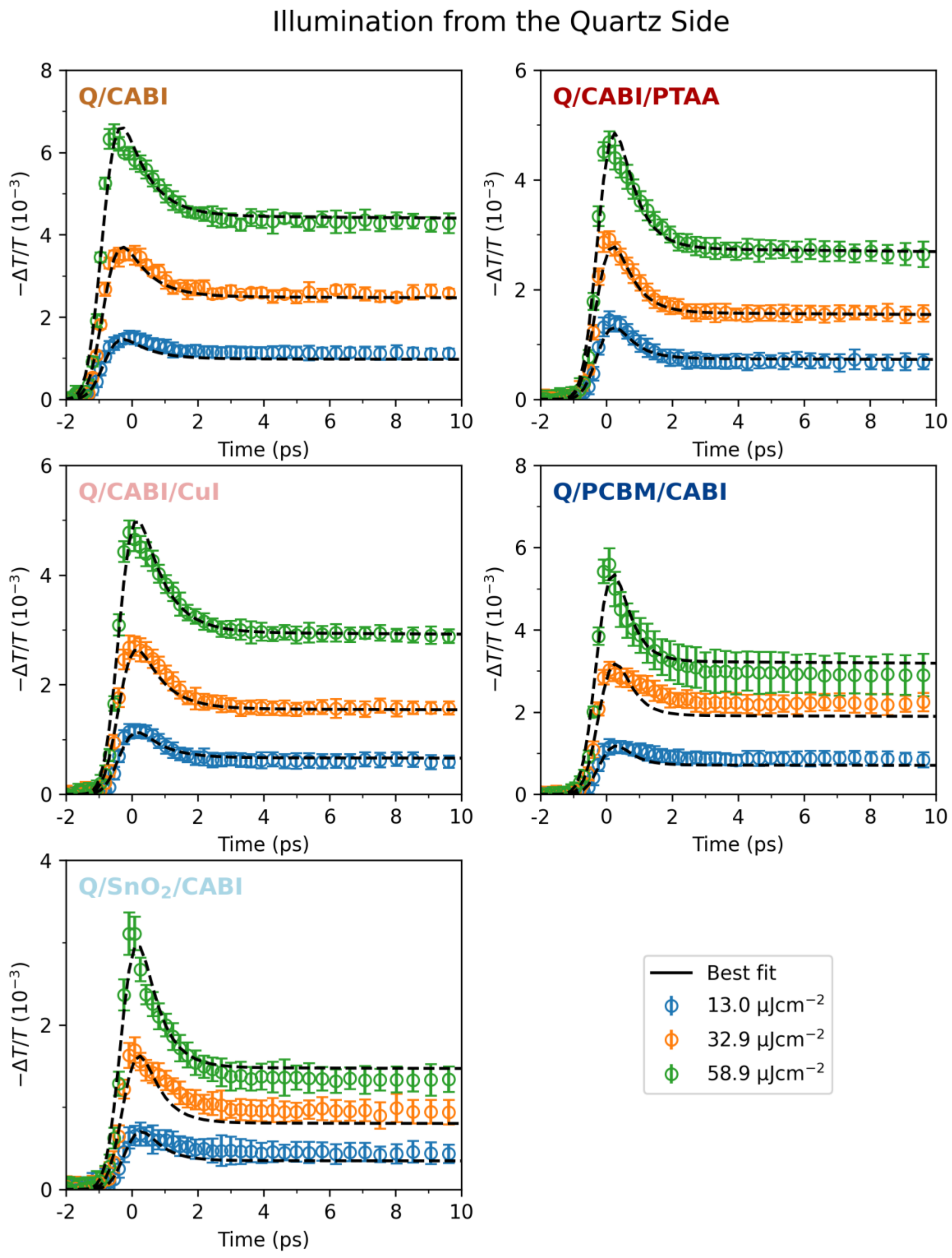

Figure S19 OPTPS photoconductivity transients shown for times between -2 and 10 ps after excitation for Cu_2AgBiI_6 thin films and half stacks with hole transport layers (PTAA and CuI) and electron transport layers (PCBM and SnO_2), deposited on a quartz substrate. Samples were illuminated from the quartz side with excitation pump fluences of 13.0, 32.9 and 58.9 μJcm^{-2}. The dotted black lines are global fits to the fluence-dependent OPTPS transients using the localization model described in Section S4.1.

S4.2 Charge-carrier Recombination After Ultrafast Localization Process

After the initial localization process, charge carriers undergo sub-nanosecond-timescale recombination as shown below. To describe the charge-carrier recombination processes in the later decay, the well-established k_1, k_2, k_3 model was used[12]. We limit the model to monomolecular and bimolecular recombination processes and assume that Auger recombination is negligible since the fluences used in our measurements are in the range of tens of uJcm^{-2}. The resulting recombination dynamics are then described by the following rate equation:

$$\frac{dn}{dt} = -k_1 n - k_2 n^2 \tag{S13}$$

Where n is the charge-carrier density, k_1 is the monomolecular recombination constant, and k_2 is the bimolecular recombination constant.

Equation S13 is first solved in terms of the experimentally observed quantity $x(t) = \Delta T/T$, which is the photoinduced change in THz transmission. $x(t)$ is proportional to $n(t)$ with the following relationship:

$$n(t)^i = \varphi^{i-1} C^{i-1} x(t)^i \tag{S14}$$

where i is the recombination order, φ is the photon-to-charge branching ratio, $C = \tilde{n}_0/x(0)$ is the proportionality factor between the immediate THz response at $t = 0$ and the absorbed photon density $\tilde{n}_0$, expressed as:

$$\tilde{n}_0 = \frac{E\lambda\, \alpha(\lambda)}{hc\, A_{eff}}\left(1 - R_{Pump}\right) \tag{S15}$$

By substituting Equation S14 into Equation S13, Equation S13 can be written as :

$$\frac{dx(t)}{dt} = -k_1 x(t) - k_2 \varphi C x(t)^2 \tag{S16}$$

The numerical solutions to this ODE are fitted globally to the decays using the least squares method across all fluences in order to extract φk_2. Given that $0 \leq \varphi \leq 1$, the values presented for k_2 reported here are underestimates for the true intrinsic values.

In order to account for an initially spatially varying charge-carrier density (due to absorption following the Beer-Lambert law), the fitting algorithm takes into account an exponentially decaying charge-carrier density. This is done by dividing the film into 30 equally thick slices and computing the decay function

for each of these individually.

Finally, the resultant average recombination rate was determined as $k_R = k_1 + k_2 n$ at each excitation fluence.

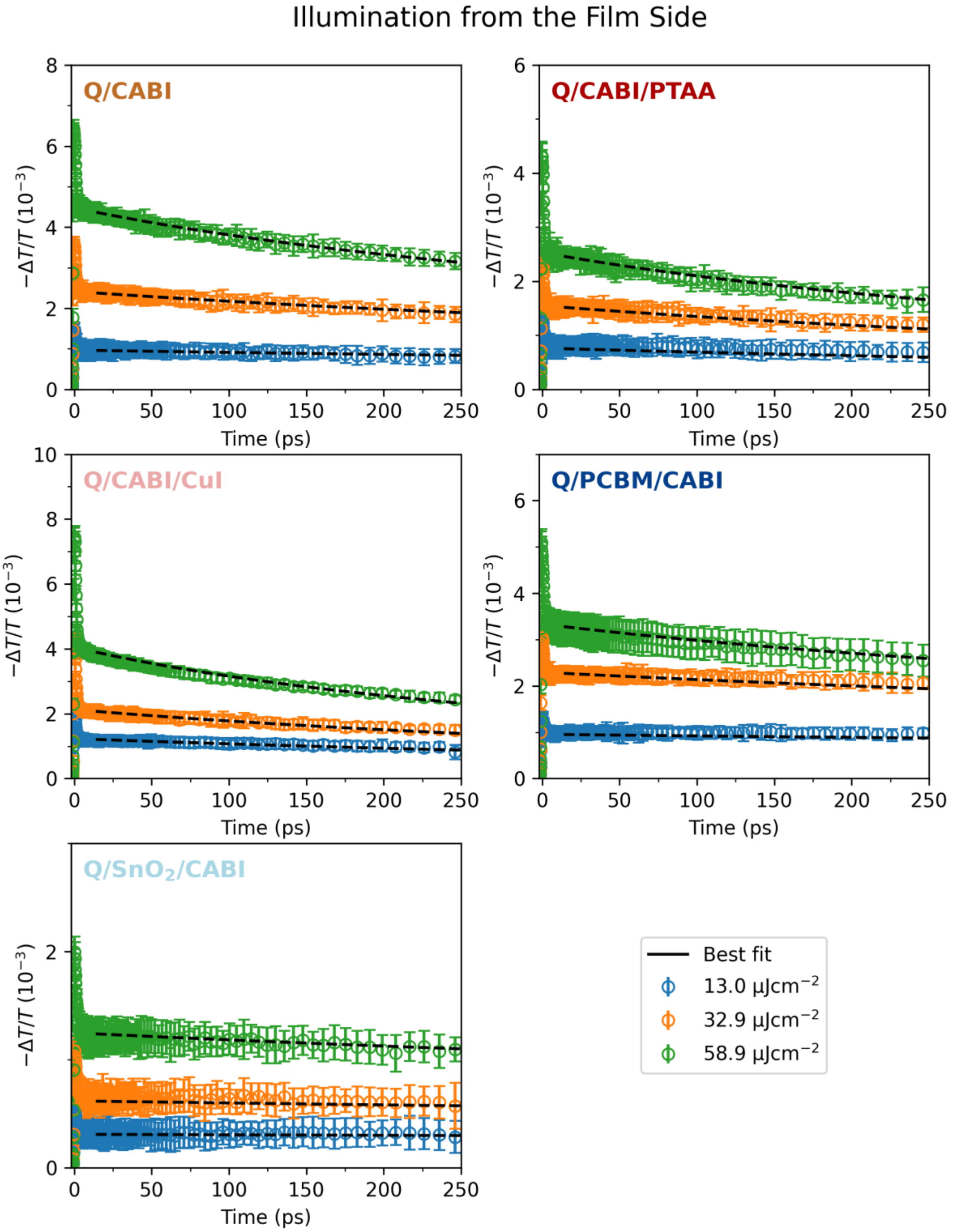

Figure S20 OPTPS photoconductivity transients for time up to 250 ps after photoexcitation for Cu_2AgBiI_6 thin films and half stacks with hole transport layers (PTAA and CuI) and electron transport layers (PCBM and SnO_2), deposited on a quartz substrate. Samples were illuminated from the film side (i.e. the side opposite the quartz substrate) with excitation pump fluences of 13.0, 32.9 and 58.9 μJcm^{-2}. The dotted black lines are global fits to the OPTPS transients of the subsequent slow decays using the k_1-k_2 model described in Section S4.2.

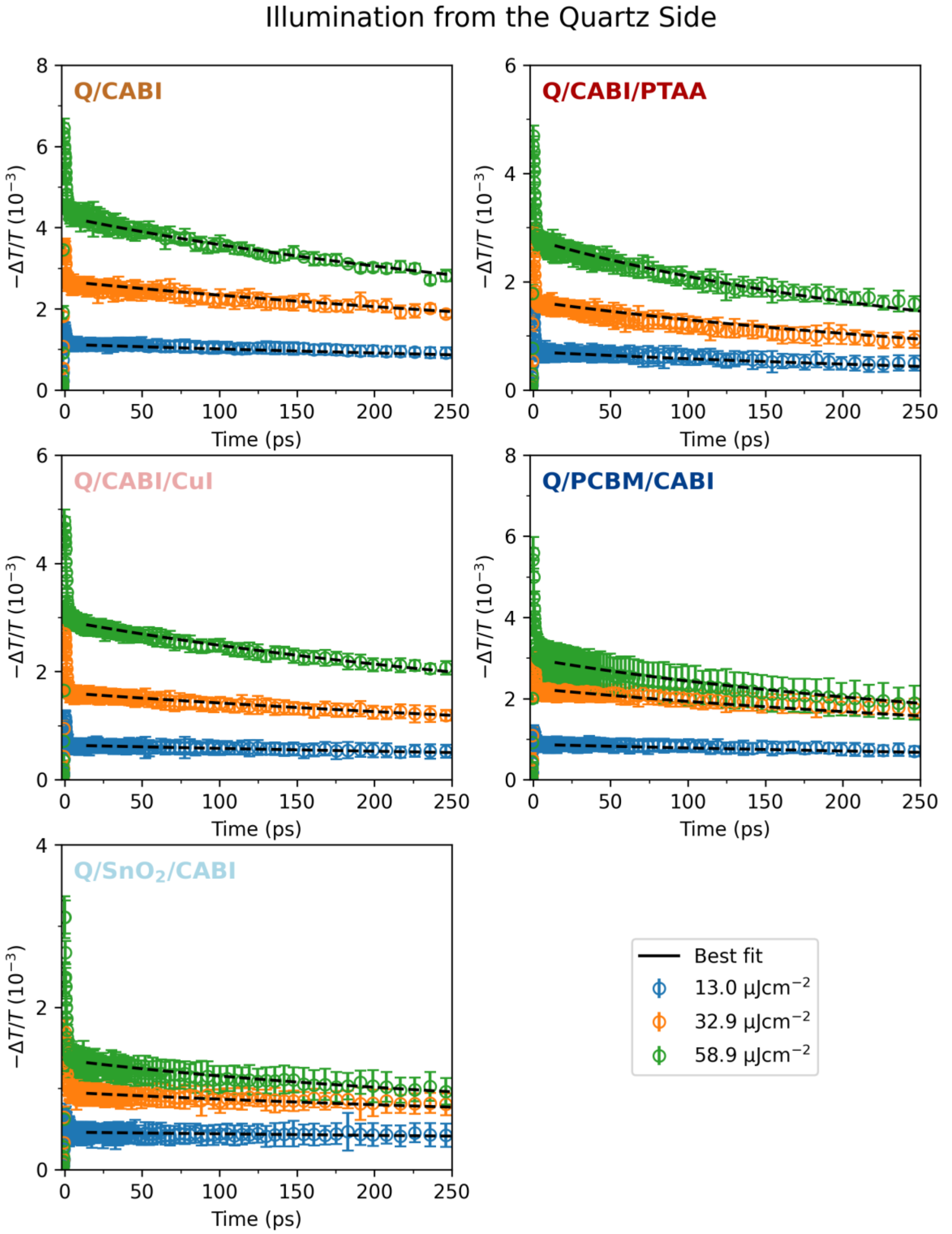

Figure S21 OPTPS photoconductivity transients for times up to 250 ps after photoexcitation for Cu_2AgBiI_6 thin films and half stacks with hole transport layers (PTAA and CuI) and electron transport layers (PCBM and SnO_2), deposited on a quartz substrate. Samples were illuminated from the film side with excitation pump fluences of 13.0, 32.9 and 58.9 μJcm^{-2}. The dotted black lines are global fits to the OPTPS transients of the subsequent slow decays using the k_1-k_2 model described in Section S4.2.

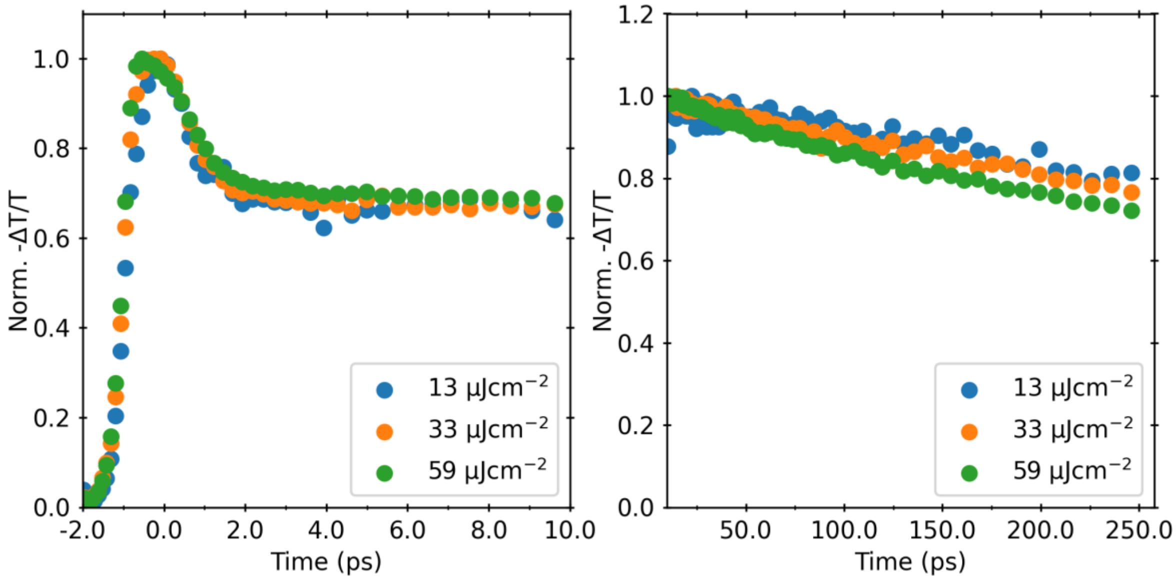

Figure S22 Normalised OPTPS early-time dynamics (from -2 ps to 10 ps) and late dynamics (from 10 ps to 250 ps) for the Q/CABI sample illuminated from the film side.

Table S4 THz mobilities and recombination rate constants obtained from the localization and k_1-k_2 model for the Cu_2AgBiI_6 thin film deposited on a quartz substrate and those interfaced with charge transport layers excited either from the film or quartz side.

Sample	Illumination Side	μ_{deloc} ($cm^2V^{-1}s^{-1}$)	μ_{loc} ($cm^2V^{-1}s^{-1}$)	k_{loc} (10^{12} s^{-1})	k_1 (10^8 s^{-1})	k_2 (10^{-11} cm^3s^{-1})
Q/CABI	Film	3.46(3)	1.655(4)	1.06(3)	7.6(5)	2.12(7)
	Quartz	3.63(7)	1.669(7)	1.31(6)	8.4(3)	2.46(8)
Q/CABI/PTAA	Film	2.61(4)	0.960(4)	1.28(4)	6.9(5)	3.3(1)
	Quartz	3.24(8)	1.029(5)	1.77(8)	16.2(6)	3.5(1)
Q/CABI/CuI	Film	4.61(5)	1.481(5)	1.20(2)	9.9(4)	3.89(8)
	Quartz	3.23(5)	1.220(5)	1.35(5)	7.6(4)	2.55(4)
Q/PCBM/CABI	Film	2.73(7)	1.191(7)	1.21(7)	1.0(3)	3.28(9)
	Quartz	3.8(2)	1.190(7)	2.2(2)	7.3(4)	4.4(1)
Q/SnO_2/CABI	Film	1.08(2)	0.450(3)	1.18(6)	0.0(8)	1.17(7)
	Quartz	2.14(8)	0.573(5)	1.7(1)	0.9(6)	3.54(8)

Owing to the impurity phases observed in the XRD patterns especially for the Q/CABI/CuI and Q/SnO_2/CABI thin films, we note that the rate constants k_{loc}, k_1 and k_2 cannot be exclusively assigned to recombination processes in CABI. Likewise, the mobilities might to some extent be affected by the impurity phases. Therefore, μ_{deloc} and μ_{loc} are treated as effective mobilities accounting for the impurity phases in the thin films as well. As discussed in the main text, the discrepancy of the mobilities between the Q/CABI thin film and other CABI thin films interfaced with charge transport layers allows us to infer information on the impurity phases, complementing the results obtained from absorption and XRD patterns.

S4.3. Instrument Response of OPTPS

The instrument response function (IRF) of our THz OPTP spectroscopy system with the 530 nm excitation wavelength was determined by measuring the finely spaced photoconductivity onset of silicon. The onset was fitted with an error function, which is defined as:

$$F(x) = A(1 - Erf\big(B(x - C)\big) + D \tag{S17}$$

Where A, B, C, D are fitting constants. The $Erf(x)$ is defined as:

$$Erf(x) = \frac{2}{\sqrt{\pi}}\int_0^x e^{-t^2}dt \tag{S18}$$

Then, the error function was converted into a Gaussian function to simulate the IRF:

$$G(x) = \alpha e^{-\left(\frac{(x-\beta)}{\sqrt{2}\sigma_{IRF}}\right)^2} \tag{S19}$$

where α is the amplitude, β is the position of the centre of the peak. σ_{IRF} was determined from the fitted value B by $\sigma_{IRF} = 1/(\sqrt{2}B)$.

From the fit, the σ_{IRF} was determined as 0.34 ps, which is faster than the timescales of the ultrafast localization process.

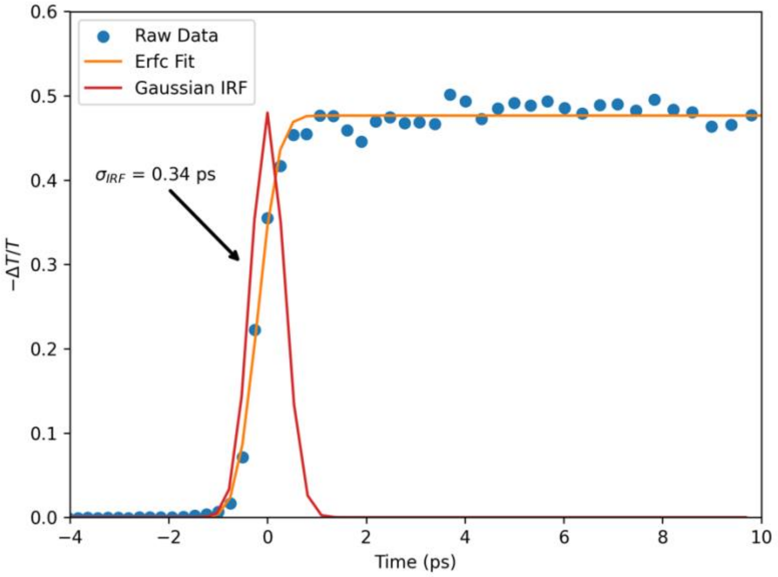

Figure S23 OPTPS photoconductivity onset of a 2 mm-thick high-resistivity silicon wafer excited at 530 nm wavelength at a fluence of 58.9 μJcm^{-2}. The experimental data were fitted with an error function in order to extract the instrument response function (IRF). The resulting Gaussian IRF has a sigma of 0.34 ps, which is much shorter than the ultrafast localization exhibited by our thin CABI films.

References

(1) Elliott, R. J. Intensity of Optical Absorption by Excitons. *Phys. Rev.* **1957**, *108*, 6. DOI: 10.1103/PhysRev.108.1384.

(2) Elmestekawy, K. A.; Wright, A. D.; Lohmann, K. B.; Borchert, J.; Johnston, M. B.; Herz, L. M. Controlling Intrinsic Quantum Confinement in Formamidinium Lead Triiodide Perovskite through Cs Substitution. *ACS Nano* **2022**, *16* (6), 9640-9650. DOI: 10.1021/acsnano.2c02970.

(3) Davies, C. L.; Filip, M. R.; Patel, J. B.; Crothers, T. W.; Verdi, C.; Wright, A. D.; Milot, R. L.; Giustino, F.; Johnston, M. B.; Herz, L. M. Bimolecular recombination in methylammonium lead triiodide perovskite is an inverse absorption process. *Nat. Commun.* **2018**, *9*, 293. DOI: 10.1038/s41467-017-02670-2.

(4) Sansom, H. C.; Longo, G.; Wright, A. D.; Buizza, L. R. V.; Mahesh, S.; Wenger, B.; Zanella, M.; Abdi-Jalebi, M.; Pitcher, M. J.; Dyer, M. S.; et al. Highly Absorbing Lead-Free Semiconductor Cu_2AgBiI_6 for Photovoltaic Applications from the Quaternary CuI–AgI–BiI_3 Phase Space. *J. Am.Chem. Soc.* **2021**, *143* (10), 3983-3992. DOI: 10.1021/jacs.1c00495.

(5) Lal, S.; Righetto, M.; Putland, B. W. J.; Sansom, H. C.; Motti, S. G.; Jin, H.; Johnston, M. B.; Snaith, H. J.; Herz, L. M. The Role of Chemical Composition in Determining the Charge-Carrier Dynamics in $(AgI)_x(BiI_3)_y$ Rudorffites. *Adv. Funct. Mater.* **2024**, *34* (32), 2315942. DOI: 10.1002/adfm.202315942.

(6) Savill, K. J.; Ulatowski, A. M.; Farrar, M. D.; Johnston, M. B.; Snaith, H. J.; Herz, L. M. Impact of Tin Fluoride Additive on the Properties of Mixed Tin-Lead Iodide Perovskite Semiconductors. *Adv. Funct. Mater.* **2020**, *30* (52), 2005594. DOI: 10.1002/adfm.202005594.

(7) Buizza, L. R. V.; Wright, A. D.; Longo, G.; Sansom, H. C.; Xia, C. Q.; Rosseinsky, M. J.; Johnston, M. B.; Snaith, H. J.; Herz, L. M. Charge-Carrier Mobility and Localization in Semiconducting Cu_2AgBiI_6 for Photovoltaic Applications. *ACS Energy Lett.* **2021**, *6*, 1729-1739. DOI: 10.1021/acsenergylett.1c00458.

(8) Putland, B. W. J.; Righetto, M.; Jin, H.; Fischer, M.; Ramadan, A. J.; Zaininger, K.-A.; Herz, L. M.; Sansom, H. C.; Snaith, H. J. Compositional Transformation and Impurity-Mediated Optical Transitions in Co-Evaporated Cu_2AgBiI_6 Thin Films for Photovoltaic Applications. *Adv. Energy Mater.* **2024**, *14* (8), 2303313. DOI: 10.1002/aenm.202303313.

(9) Xia, C. Q.; Peng, J.; Poncé, S.; Patel, J. B.; Wright, A. D.; Crothers, T. W.; Rothmann, M. U.; Borchert, J.; Milot, R. L.; Kraus, H.; et al. Limits to Electrical Mobility in Lead-Halide Perovskite Semiconductors. *J. Phys. Chem. Lett.* **2021**, *12* (14), 3607-3617. DOI: doi.org/10.1021/acs.jpclett.1c00619.

(10) Righetto, M.; Wang, Y.; Elmestekawy, K. A.; Xia, C. Q.; Johnston, M. B.; Konstantatos, G.; Herz, L. M. Cation-Disorder Engineering Promotes Efficient Charge-Carrier Transport in $AgBiS_2$ Nanocrystal Films. *Adv. mater.* **2023**, *35* (48), 2305009. DOI: 10.1002/adma.202305009.

(11) Lee, J. E.; Motti, S. G.; Oliver, R. D. J.; Yan, S.; Snaith, H. J.; Johnston, M. B.; Herz, L. M. Unraveling Loss Mechanisms Arising from Energy-Level Misalignment between Metal Halide Perovskites and Hole Transport Layers. *Adv. Funct. Mater.* **2024**, *34* (30), 2401052. DOI: 10.1002/adfm.202401052.

(12) Herz, L. M. Charge-Carrier Dynamics in Organic-Inorganic Metal Halide Perovskites. *Rev. Phys. Chem.* **2016**, *67*, 65-89. DOI: 10.1146/annurev-physchem-040215-112222.
